# ﻿Complete mitogenomes of four *Trichiurus* species: A taxonomic review of the *T.lepturus* species complex

**DOI:** 10.3897/zookeys.1084.71576

**Published:** 2022-01-26

**Authors:** Mu-Rong Yi1, Kui-Ching Hsu*, Sui Gu, Xiong-Bo He, Zhi-Sen Luo, Hung-Du Lin*, Yun-Rong Yan*

**Affiliations:** 1 College of Fisheries, Guangdong Ocean University, Zhanjiang 524088, China; 2 Marine Resources Big Data Center of South China Sea, Southern Marine Science and Engineering Guangdong Laboratory, Zhanjiang 524088, China; 3 The Affiliated School of National Tainan First Senior High School, Tainan 701, Taiwan; 4 Guangdong Provincial Engineering and Technology Research Center of Far Sea Fisheries Management and Fishing of South China Sea, Guangdong Ocean University, Zhanjiang 524088, China

**Keywords:** Characterization, mitogenome, molecular tool, phylogeny, taxonomy, *Trichiurus*

## Abstract

Four *Trichiurus* species, *T.japonicus*, *T.lepturus*, *T.nanhaiensis*, and *T.brevis*, from the coasts of the China Seas, have been identified and their entire mitochondrial genomes (mitogenomes) have been sequenced by next-generation sequencing technology. A comparative analysis of five mitogenomes was conducted, including the mitogenome of *T.gangeticus*. The mitogenomes contained 16.568–16.840 bp and encoded 36 typical mitochondrial genes (13 protein-coding, 2 ribosomal RNA-coding, and 21 transfer RNA-coding genes) and two typical noncoding control regions. Although tRNA^Pro^ is absent from *Trichiurus* mitogenomes, when compared with the 22 tRNAs reported in other vertebrates, the gene arrangements in the mitogenomes of the studied species are consistent with those in most teleost mitogenomes. The full-length sequences and protein-coding genes (PCGs) in the mitogenomes of the five species had obvious AT biases and negative GC skew values. Our study indicate that the specimens in the Indian Ocean are neither *T.lepturus* nor *T.nanhaiensis* but they are *T.gangeticus*; the *Trichiurus* species composition in the Indian Ocean is totally different from that in Pacific and Atlantic oceans; there are at least two *Trichiurus* species in Indian Ocean; and the worldwide systematics and diversity of the genus *Trichiurus* need to be reviewed.

## ﻿Introduction

The cutlassfishes include ten genera and 47 species in Eschmeyer’s Catalog of Fishes (ECoF, Fricke et al. 2021). These species are predatory fishes in the family Trichiuridae (Scombriformes) and found in seas throughout the world (Nelson et al. 2016). Among the ten genera, members of the genus *Trichiurus* Linnaeus, 1758 are the most common and most well studied. *Trichiurus* species are important commercial marine fishes (FAO 2004); however, their systematics remain unresolved because of the high degree of similarity among species in the genus in terms of bodily appearance and silvery coloration. As many as 31 nominal species of the genus *Trichiurus* have been described to date, but only nine are valid species (FishBase, Froese and Pauly 2021). However, according to ECof, *Trichiurus* has 31 nominal names and eleven valid species. The difference between the two databases is due to *T.japonicus* Temminck & Schlegel, 1844 and *T.nitens* Garman, 1899. FishBase considers these two species to be synonymous with *T.lepturus*, based on Nakamura and Parin (1993). However, Chakraborty et al. (2006a) established that *T.japonicus* is a valid species based on the differences in mitochondrial 16S rRNA. Moreover, Burhanuddin and Parin (2008) proved the validity of *T.nitens* based on the morphometric parameters.

According to ECoF, these eleven valid species are divided between two species complexes, the *T.lepturus* complex and the *T.russelli* complex. The *T.lepturus* complex is referred to as the large-headed or long-tailed species complex. This species complex, which has the anal opening positioned vertically at the 38^th^–41^st^ dorsal fin rays, includes seven species: *T.lepturus* Linnaeus, 1758, *T.japonicus*, *T.auriga* Klunzinger, 1884, *T.nitens*, *T.gangeticus* Gupta, 1966, *T.margarites* Li, 1992 and *T.nanhaiensis* Wang & Xu, 1992. The *T.russelli* complex is referred to as the short-tailed species complex, and the anal opening is positioned vertically at the 34^th^ and 35^th^ dorsal fin rays (Burhanuddin et al. 2002). The short-tailed species complex includes four species: *T.australis* Chakraborty, Burhanuddin & Iwatsuki, 2005, *T.brevis* Wang & You, 1992, *T.nickolensis* Burhanuddin & Iwatsuki, 2003 and *T.russelli* Dutt & Thankam, 1967. Although there were many studies about the systematics of the genus *Trichiurus* (e.g., Lee et al. 1977; Nakabo 2000; Chakraborty et al. 2006b; Tzeng et al. 2007; Hsu et al. 2009), the taxonomic identification within the *T.lepturus* complex has long been confusing.

Many studies have suggested that *Clupeahaumela* Fabricius, 1775 is a synonym of *T.lepturus* (Nakamura and Parin 1993, 2021; Fricke 2008; Golani and Fricke 2018); however, a recently published study (Zheng et al. 2019) mentioned this species as a valid *Trichiurus* species without taxonomic evidence and presented its complete mitochondrial genome. In addition, many studies (Tucker 1956; Nakamura and Parin 1993; Nelson 1994) suggested that *T.japonicus* Temminck & Schlegel, 1844 is synonymous with *T.lepturus*, but other studies (Lee et al. 1977; Nakabo 2000; Chakraborty et al. 2006a, b; Tzeng et al. 2007; Hsu et al. 2009; He et al. 2014; Fricke et al. 2021) suggested that *T.japonicus* is a valid species. *Trichiuruslepturus* is known to be found in tropical and temperate waters throughout the world (Froese and Pauly 2021). Chakraborty et al. (2006a) sampled specimens of *T.lepturus* in the Indian Ocean, but Hsu et al. (2009) re-examined the taxonomic status of *Trichiurus* species and suggested that these specimens from the Indian Ocean might not be *T.lepturus*. There are thus several outstanding questions regarding the systematics and distributional patterns of *Trichiurus* species.

The accurate identification of species is important both for scientists and the broader community. However, correctly identifying species remains a major challenge for the general public. Hebert et al. (2003) proposed that the DNA barcoding can be used to facilitate species identification. For animals, the universal barcoding region is the cytochrome c oxidase subunit 1 (COI) in mitochondrial DNA. COI has become a valuable molecular tool for studies characterizing interspecific and intraspecific diversity and evolutionary relationships (e.g., Conway et al. 2015; Ahti et al. 2016; Salcioglu et al. 2020). However, Mirande (2018) proposed that incomplete mitochondrial gene sequences have a limited ability to facilitate the identification of complex evolutionary relationships in many fishes. The use of mitogenomes would be expected to provide more information for species identification, phylogenetics and population genetics (Liu et al. 2020; Phillips and Zakaria 2021; Wang et al. 2021). To address these problems about the taxonomy of the genus *Trichiurus*, the COI, mitogenome, and morphology were used.

In this study, we completed four tasks. First, COI sequences were used to identify *Trichiurus* species to determine the number of species found along the coast of China. Second, the complete mitogenomes of four *Trichiurus* species in the China Seas were sequenced using next-generation sequencing. Third, we obtained the mitogenome sequences of the family Trichiuridae from the NCBI database (https://www.ncbi.nlm.nih.gov) to clarify the systematics of the genus *Trichiurus* and to facilitate comparison of the molecular evolutionary characteristics between *Trichiurus* species and other cutlassfishes. Finally, traditional caliper measurements were performed, which identified 14 landmarks that were used to evaluate morphological differences among *Trichiurus* species. These results provide further insight into the systematics and diversity of the genus *Trichiurus*.

## ﻿Materials and methods

### ﻿Sampling and species identification

**Figure 1.**
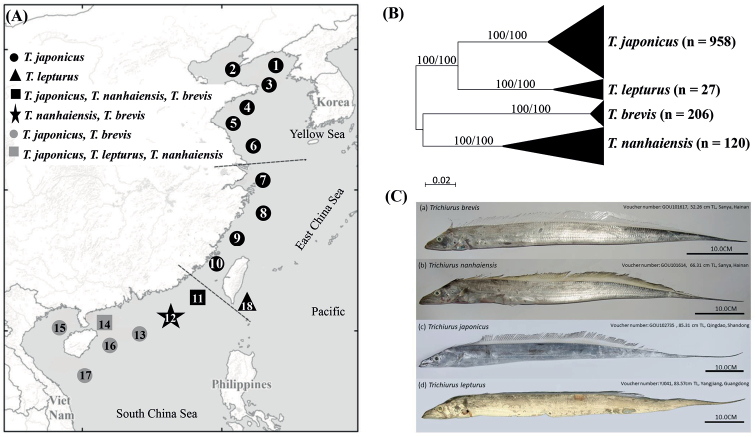
**A** Eighteen sampling localities of the genus the *Trichiurus* along the Chinese coast and the species composition after our surveys. Refer to Suppl. material 1: Table S1 for the abbreviations of localities. **B** The maximum-likelihood (ML) tree of these four *Trichiurus* species along the coast based on the COI gene. The numbers at the nodes are bootstrap values of the ML and NJ (neighbor-joining) analyses. The sampling size (n) indicated in parentheses **C** The photographs of four Trichiurus species used in the mitogenomes analyses.

**Figure 2.**
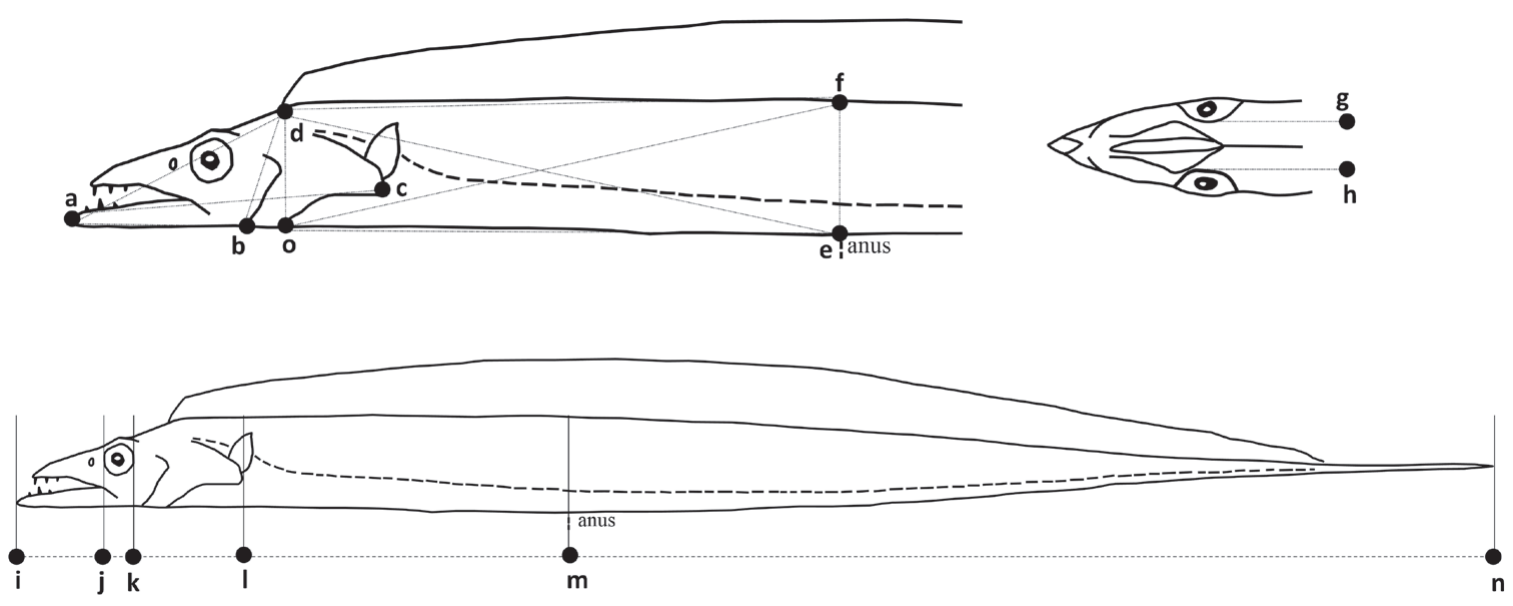
Positions of 14 (a–n) landmarks used to contrast the morphological differences between *Trichiurus* species.

Our teams sampled *Trichiurus* specimens from the China Seas, including the Yellow Sea, East China Sea, and South China Sea in October 2017 and August 2019 by longline, gill net, and trawl net with fishermen (Fig. 1A, Suppl. material 1: Table S1). In total, 1.311 specimens were collected. Traditional caliper measurements were performed, which identified 14 landmarks (a–n, Fig. 2).

A portion of the muscle tissues from 1.311 specimens was stored in 100% ethanol. Total genomic DNA was extracted from muscle tissue using a Genomic DNA Purification Kit (Gentra Systems, Valencia, CA). The COI gene was amplified by polymerase chain reaction (PCR) using the primers Fish-F2 (5´-ACCTCTGTGTGTGGGGCTAC-3´) and Fish-R2 (5´-GTGATGCATTGGCTTGAAA-3´) (Gu et al. 2021). Each 50-µl PCR mixture contained 5 ng of template DNA, 5 µl of 10× reaction buffer, 4 µl of dNTP mix (10 mM), 5 pmol of each primer and 2 U of Taq polymerase (TaKaRa, Taq polymerase). PCR was conducted on an MJ Thermal Cycler using the following cycling parameters: one cycle of denaturation at 94 °C for 3 min, 40 cycles of denaturation at 94 °C for 30 s, annealing at 55 °C for 30 s and extension at 72 °C for 1 min and 30 s, followed by a 72 °C extension for 10 min and storage at 4 °C. The purified PCR products were sequenced using an ABI 377 automated sequencer (Applied Biosystems, Foster City, CA, U.S.A.). The resulting chromatograms were assessed using CHROMAS software (Technelysium), and the sequences were manually edited using BIOEDIT 6.0.7 (Hall 1999). In totally, 1.311 sequences were obtained, and the haplotypes were deposited in GenBank under accessions MZ959870 - MZ959999, MZ960057-MZ960127, OK053821 - OK054341 and OL539388-OL539398. The nucleotide sequences were aligned in Clustal X 1.81 (Thompson et al. 1997). Selection of the best-fit nucleotide substitution models was performed using the Bayesian information criterion (BIC) in jModelTest 2.0 (Darriba et al. 2012). The most appropriate nucleotide substitution model was GTR+I+G for COI. Maximum likelihood (ML) and neighbor-joining (NJ) phylogenetic analysis were performed with MEGA-X (Kumar et al. 2018). Bootstrapping was implemented with 1000 replications. In addition, Shen et al. (2016) proposed that the use of the K2P (Kimura’s two-parameter) distance in barcode analyses has been challenged and the p-distance has been proposed to be a better model. Thus, the p-distances between *Trichiurus* species were estimated in MEGA-X.

### ﻿Sequence assembly, annotation, and analysis

Next-generation sequencing (NGS) was performed to obtain complete mitogenome sequences. Complete mitogenomes were obtained from high-throughput sequencing with a HiSeqX Ten platform (Illumina, San Diego, CA) with a paired-end, 150-bp approach. All the reads were mapped to the full mitogenome reference sequences of other *Trichiurus* species (Table 1) using SOAPdenovo v.2.04 (https://github.com/aquaskline/SOAPdenovo2). The remaining high-quality reads were assembled using SPAdes v3.10 (https://github.com/ablab/spades). Compared with the corresponding complete mitogenome sequences of the genus *Trichiurus* (Liu and Cui 2009; Liu et al. 2013; Xu et al. 2019; Zheng et al. 2019; Mukundan et al. 2020; Table 1), protein-coding genes (PCGs), tRNA-coding genes and ribosome-coding genes were identified by BLAST. Codon usage, nucleotide substitution and base composition were determined using MEGA-X and DnaSP version 5.10 (Librado and Rozas 2009), and the rules for the vertebrate mitochondrial genetic code was used. AT skewing and GC skewing of the nucleotide composition were measured according to the following formulae: AT skew = (A – T)/(A + T) and GC skew = (G – C)/(G + C) (Perna and Kocher 1995).

**Table 1.** Information on the mitogenomes used in this study.

Species	Accession no.	Genome size	References
*Trichiurusjaponicus*	EU339148	16.796 bp	Liu and Cui (2009)
	MK292708	16.798 bp	Xu et al. (2019)
	MW719077	16.685 bp	This study
*T.haumela*	MH846121	16.855 bp	Zheng et al. (2019)
*T.lepturus*	MK333401	16.840 bp	Mukundan et al. (2020)
*T.nanhaiensis*	MW719078	16.568 bp	This study
	JX477078	17.060 bp	Liu et al. (2013)
	MW719076	16.801 bp	This study
*T.brevis*	MW694877	16.733 bp	This study
*Benthodesmustenuis*	AP012522	16.864 bp	Miya et al. (2013)
*Aphanopuscarbo*	AP012944	16.406 bp	Miya et al. (2013)
*Evoxymetoponpoeyi*	AP012509	16.475 bp	Miya et al. (2013)
*Assurgeranzac*	AP012508	16.510 bp	Miya et al. (2013)

The relative synonymous codon usage (RSCU), nonsynonymous codon usage (Ka) and synonymous codon usage (Ks) of all PCGs were analyzed using DnaSP. Comparison of the rates of Ka/Ks provides insight into changes in selective pressure: Ka/Ks values > 1 indicate positive selection; Ka/Ks = 1 indicates neutral selection; and Ka/Ks < 1 indicates negative or purifying selection. Some mitogenomes of the family Trichiuridae were downloaded from GenBank (NCBI database, Table 1). The most appropriate nucleotide substitution model was GTR+I+G for the mitogenome. The ML and NJ phylogenetic analysis were performed with MEGA-X. Bootstrapping was implemented with 1000 replications. The p-distances between *Trichiurus* species (interspecific) and between genera within Trichiuridae (intergeneric) were estimated in MEGA-X.

### ﻿Morphological analyses

Measurements were referred to the truss network (Humphries et al. 1981) and some additional landmarks, forming 19 distances from 14 landmarks (Fig. 2). The morphometric characteristics were measured to nearest 0.1 and 0.01 cm using traditional calipers. In total, 225 specimens from South China Sea were measured. Values of the distances between landmarks were measured, and their means and standard deviations (S.D.) were calculated.

## ﻿Results

### ﻿Species identification

A total of 1.311 specimens were collected. Species were first identified by morphology. Two species groups were recognized, the *T.lepturus* complex, which has the anal opening positioned vertically at the 38^th^–41^st^ dorsal fin rays, and the *T.russelli* complex, which has the anal opening positioned vertically at the 34^th^ and 35^th^ dorsal fin rays (Burhanuddin et al. 2002). Within the *T.lepturus* complex, *T.japonicus* has a longer tail, and *T.lepturus* has a whitish dorsal fin when fresh; by contrast, *T.nanhaiensis* has a yellowish green dorsal fin (Hsu et al. 2009). Besides, our study found that from the front view of the heads preserved specimens, the frontal bone of *T.nanhaiensis* is very smooth (Suppl. material 1: Fig. S1A), the frontal bone of *T.japonicus* is slightly inverted (Suppl. material 1: Fig. S1B), and the frontal bone of *T.lepturus* is obviously inverted and bulges in the upper part of the orbit and is accompanied by an indentation (Suppl. material 1: Fig. S1C). Four species belonging to the two species complexes were collected. We used COI sequences to identify species (Hebert et al. 2003). Our study sequenced complete COI gene (1551 bp) in all specimens. The phylogenetic trees reconstructed within ML and NJ were identical. In the ML tree (Fig. 1B), all specimens were grouped into four lineages with strong bootstrap support. After BLAST, we ensured these four lineages corresponded to four *Trichiurus* species: *T.japonicus* (*n* = 958), *T.lepturus* (*n* = 27), *T.nanhaiensis* (*n* = 120) and *T.brevis* (*n* = 206).

*Trichiurusjaponicas* is distributed in the China Sea; *T.lepturus*, *T.nanhaiensis*, and *T.brevis* are distributed in the South China Sea. The results from the morphological and molecular data were the same. However, our study revealed that *T.lepturus* is very rare in the South China Sea (Fig. 1, Suppl. material 1: Table S1). Additionally, the results showed that *T.lepturus* complex was not a monophyletic group because *T.brevis*, belonging to *T.russelli* complex, was nested with *T.nanhaiensis*. Our study considers that this is because information is lacking.

**Figure 3.**
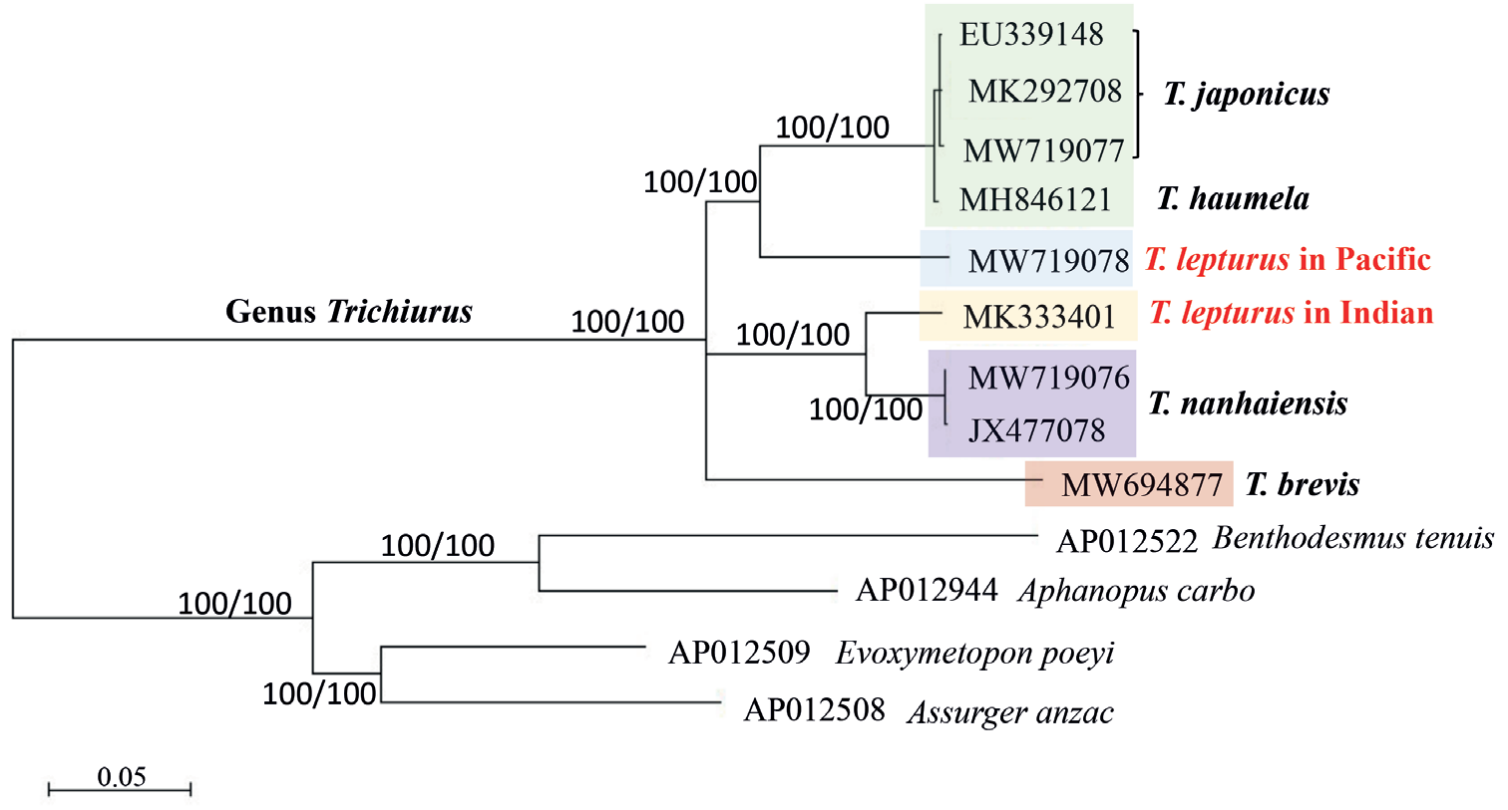
The maximum-likelihood (ML) tree of the Trichiuridae based on the sequences of mitogenome (excluding d-loop). The numbers at the nodes are bootstrap values of the ML and NJ (neighbor-joining) analyses.

**Figure 4.**
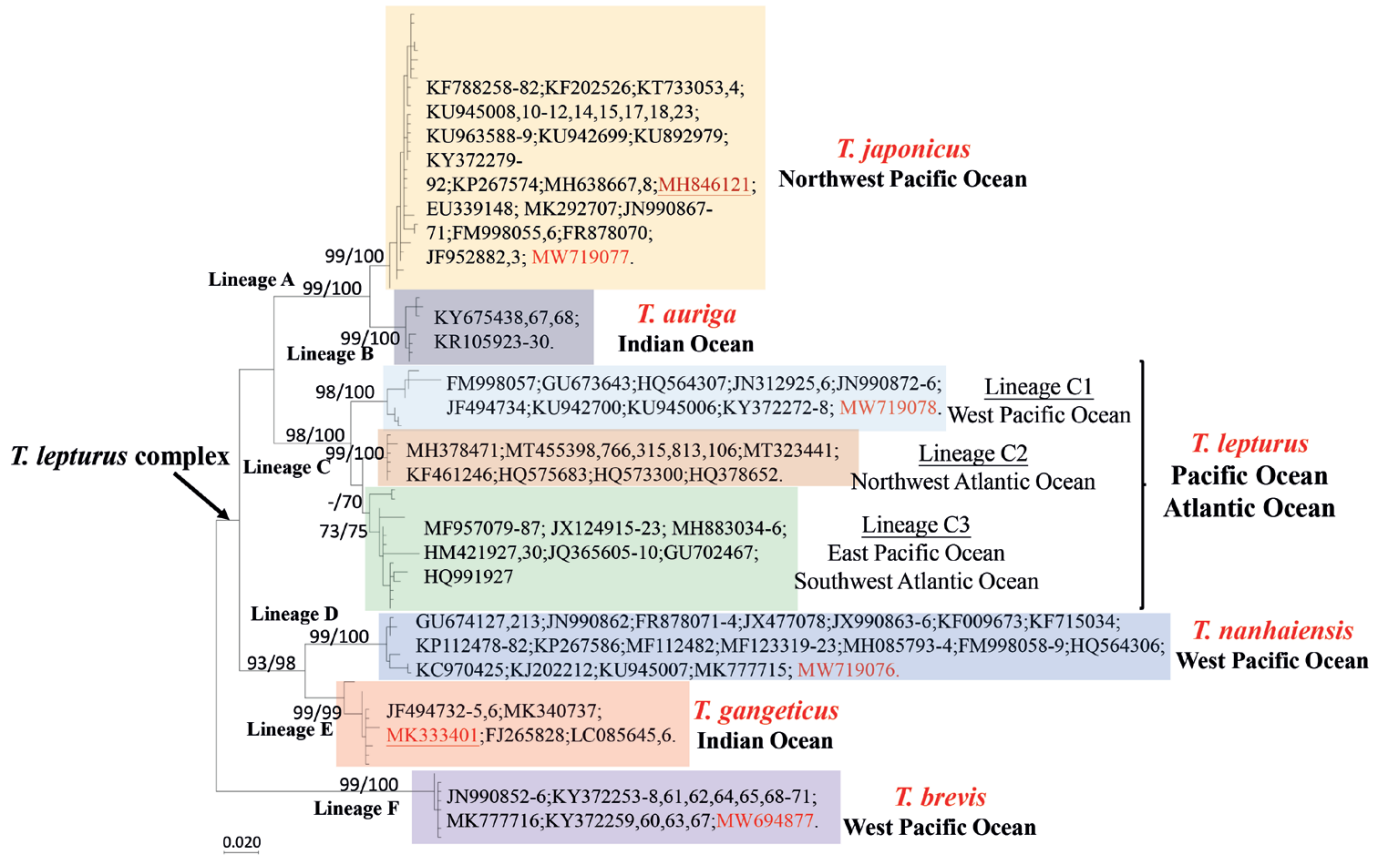
The maximum-likelihood (ML) tree of six *Trichiurus* species in the world based on the COI gene. The numbers at the nodes are bootstrap values of the ML and NJ (neighbor-joining) analyses.

After identifying species by morphology and DNA barcoding, the complete mitochondrial genomes of four *Trichiurus* species were sequenced (Fig. 1C). These four specimens were fixed in 10% formalin, transferred to 70% ethanol, and deposited in the Guangdong Ocean University, Zhanjiang, China as voucher specimens (GOU101614, GOU101617, GOU102735, and TLYJ041). The lengths of the complete mitogenomes of *T.japonicus* (MW719077), *T.lepturus* (MW719078), *T.nanhaiensis* (MW719076), and *T.brevis* (MW694877) were 16.685 bp, 16.568 bp, 16.801 bp, and 16.733 bp, respectively. To confirm the taxonomy of *Trichiurus* species, the phylogeny of Trichiuridae was analyzed using mitogenome sequences (Fig. 3, Table 1). The phylogenetic trees reconstructed within ML and NJ were identical. In ML tree (Fig. 3), the sequences of the genus *Trichiurus* were grouped into five lineages (I–V). *Trichiurushaumela* (MH846121 in Zheng et al. 2019) was included within *T.japonicus* (lineage I), and *T.lepturus* (MW719078) in our study and “*T.lepturus*” (MK333401 in Mukundan et al. 2020) were not considered monophyletic (lineages II and III). Thus, our study used COI sequences to examine the taxonomic status of *Trichiurus* species. All COI sequences of *Trichiurus* species in GenBank (NCBI database) were downloaded. After alignment, 477 bp were analyzed. The phylogenetic trees reconstructed within ML and NJ were identical, with only small differences in bootstrap values. In the COI phylogenetic analyses (ML tree, Fig. 4), all sequences were grouped into six lineages (A–F). Lineage F included *T.brevis* within the *T.russelli* complex. *Trichiurushaumela* (MH846121 in Zheng et al. 2019) was also included within *T.japonicus* in lineage A. The specimen from the Indian Ocean (MK333401 in Mukundan et al. 2020) might be not *T.lepturus*, as it was grouped with other specimens of *T.gangeticus* in lineage E. The genetic distance within the six lineages ranged from 0.0013 (lineage F, *T.brevis*) to 0.0333 (lineage C, *T.lepturus*), and the genetic distance between lineages ranged from 0.0435 (between *T.japonicus* and *T.auriga*) to 0.1600 (between *T.japonicus* and *T.brevis*) (Table 2). Based on the mitogenomes, the genetic distances between these five species ranged from 0.0507 (*T.nanhaiensis* and *T.gangeticus*) to 0.1331 (*T.gangeticus* and *T.brevis*), including the d-loop region, and from 0.0476 (*T.nanhaiensis* and *T.gangeticus*) to 0.1288 (*T.lepturus* and *T.brevis*), excluding the d-loop region (Table 2). Moreover, the mitogenome p-distances between *T.japonicus* and *T.haumela*, including and excluding the d-loop region, were 0.0067 and 0.0047, respectively.

**Table 2.** The p-distance based on sequences of partial COI (below) and mitogenome (above, excluding d-loop in brackets). Bold indicates the mean COI divergence within groups.

	*T.japonicus*	*T.auriga*	*T.lepturus*	*T.nanhaiensis*	*T.gangeticus*	*T.brevis*
*T.japonicus*	**0.0054**	–	0.0984 (0.0965)	0.1160 (0.1127)	0.1140 (0.1114)	0.1306 (0.1280)
*T.auriga*	0.0435	**0.0069**	–	–	–	-
*T.lepturus*	0.1078	0.1149	**0.0333**	0.1127 (0.1118)	0.1119 (0.1107)	0.1310 (0.1288)
*T.nanhaiensis*	0.1277	0.1171	0.1255	**0.0037**	0.0507 (0.0476)	0.1308 (0.1244)
*T.gangeticus*	0.1251	0.1156	0.1093	0.0750	**0.0090**	0.1331 (0.1279)
*T.brevis*	0.1600	0.1505	0.1475	0.1282	0.1357	**0.0013**

### ﻿Morphological analyses

**Figure 5.**
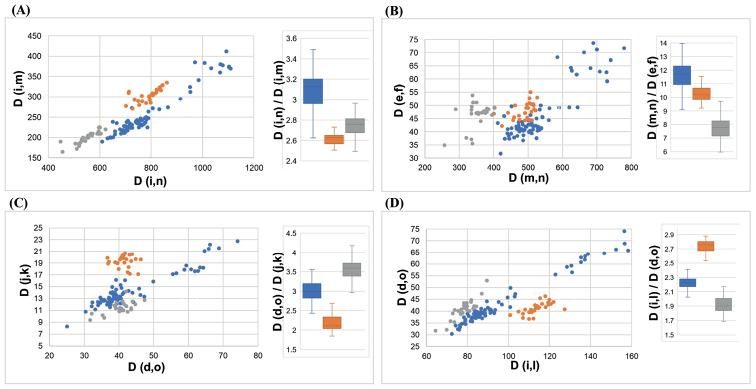
The simple regression and the boxplot analysis in *T.japonicus* (blue), *T.lepturus* (orange) and *T.nanhaiensis* (grey) **A** Total length [D(i,n)] and Preanal length [D(i,m)] **B** Caudal length [D(m,n)] and Body depth at anus [D(e,f)] **C** Head depth [D(d,o)] and Orbital length [D(j,k)] and **D** Head length [D(i,l)] and Head depth [D(d,o)]. The landmarks are illustrated in Fig. 2.

After identifying species by morphological characters and phylogenetic analysis, traditional caliper measurements were performed, which produced 14 landmark sites (a–n, Fig. 2). *Trichiurusbrevis* could not be identified by these standard morphological characteristics (Table 3), but it is easy to distinguish from *T.lepturus* complex in the anal opening positioned vertically at the 34^th^ and 35^th^ dorsal fin rays. Thus, our morphological comparison is mainly concentrated in the *T.lepturus* complex (Table 3, Fig. 5). Our study compared many numerical values based on the external morphology of various body ratios (more than 20 counts, Suppl. material 1: Fig. S2). Our study found that the caudal length is longer in *T.japonicus* [D(i,n/i,m) = 3.08 ± 0.32, 2.61 ± 0.09 and 2.74 ± 0.18 in *T.japonicus*, *T.lepturus*, and *T.nanhaiensis*; Table 3, Fig. 5A]; the body depth at the anus is wider in *T.nanhaiensis* [D(m,n/e,f) = 11.60 ± 1.79, 10.29 ± 0.87 and 7.77 ± 1.38 in *T.japonicus*, *T.lepturus*, and *T.nanhaiensis*; Table 3, Fig. 5B]; the orbital length is larger in *T.lepturus* [D(d,o/j,k) = 3.00 ± 0.42, 2.20 ± 0.32 and 3.57 ± 0.44 in *T.japonicus*, *T.lepturus*, and *T.nanhaiensis*; Table 3, Fig. 5C]; and the head is slenderer in *T.lepturus* [D(i,l/d,o) = 2.22 ± 0.14, 2.73 ± 0.13 and 1.92 ± 0.18 in *T.japonicus*, *T.lepturus* and *T.nanhaiensis*; Table 3, Fig. 5D].

**Table 3.** Summary statistics of body measurements for four *Trichiurus* species.

Measurement (cm)	Mean ± S.D.			
	*T.japonicus*	*T.lepturus*	*T.nanhaiensis*	*T.brevis*
Total length [D(i,n)*]	74.7 ± 12.8	79.8 ± 6.2	55.6 ± 9.2	50.6 ± 7.3
D(i,m)	23.7 ± 0.6	30.5 ± 0.2	20.5 ± 0.2	28.0 ± 0.3
D(i,l)	8.7 ± 2.6	11.4 ± 1.0	7.8 ± 0.8	6.4 ± 1.2
D(m,n)	50.8 ± 10.5	49.7 ± 5.0	35.5 ± 5.2	32.8 ± 4.6
D(i,j)	3.0 ± 1.1	3.8 ± 0.5	2.6 ± 0.3	2.2 ± 0.4
D(j,k)	1.3 ± 0.4	1.9 ± 0.1	1.1 ± 0.2	1.0 ± 0.1
D(k,l)	4.4 ± 1.0	5.7 ± 0.5	4.1 ± 0.4	3.3 ± 0.6
D(a,b)	5.2 ± 1.6	5.6 ± 0.7	5.3 ± 0.5	3.9 ± 1.0
D(a,c)	8.3 ± 1.8	11.0 ± 1.0	7.4 ± 0.9	6.1 ± 1.0
D(a,d)	6.0 ± 2.0	8.1 ± 0.7	5.9 ± 0.7	4.4 ± 0.7
D(b,c)	3.9 ± 1.5	5.8 ± 0.6	2.8 ± 0.4	2.5 ± 0.7
D(b,d)	3.9 ± 1.2	5.1 ± 0.5	4.0 ± 0.3	3.3 ± 0.5
D(b,e)	28.6 ± 4.8	24.5 ± 2.2	17.4 ± 1.1	13.8 ± 2.8
D(b,f)	19.2 ± 5.0	25.1 ± 1.7	18.2 ± 1.3	14.4 ± 3.0
D(c,d)	3.9 ± 0.8	4.6 ± 0.5	3.4 ± 0.5	3.1 ± 0.5
D(d,e)	18.2 ± 4.6	23.1 ± 1.9	17.5 ± 1.3	14.2 ± 2.6
D(d,f)	17.9 ± 5.8	22.7 ± 1.8	17.0 ± 1.4	13.9 ± 2.6
D(d,o)	3.8 ± 0.3	4.1 ± 0.2	4.1 ± 0.2	3.3 ± 0.3
D(e,f)	4.2 ± 0.1	4.8 ± 0.5	4.7 ± 0.4	3.6 ± 0.6
D(g,h)	1.3 ± 0.4	1.7 ± 0.3	1.0 ± 0.2	0.9 ± 0.1
D(i,n)/D(i,m)	3.08 ± 0.32	2.61 ± 0.09	2.74 ± 0.18	2.86 ± 0.11
D(m,n)/D(i,m)	2.08 ± 0.32	1.62 ± 0.09	1.74 ± 0.18	1.86 ± 0.11
D(i,m)/D(e,f)	5.59 ± 0.57	6.29 ± 0.45	4.47 ± 0.79	4.91 ± 0.43
D(m,n)/D(e,f)	11.60 ± 1.79	10.29 ± 0.87	7.77 ± 1.38	9.13 ± 0.69
D(i,l)/D(d,o)	2.22 ± 0.14	2.73 ± 0.13	1.92 ± 0.18	2.55 ± 0.13
D(d,o)/D(g,h)	3.10 ± 0.42	2.42 ± 0.36	3.88 ± 0.67	3.57 ± 0.56
D(d,o)/D(j,k)	3.00 ± 0.42	2.20 ± 0.32	3.57 ± 0.44	3.31 ± 0.41
Sample size	75	27	27	96

* D(i,n), distance between landmarks i and n in Fig. 2.

### ﻿Genome organization, base composition and rates

The mitogenomes of all four *Trichiurus* species contain 36 mitochondrial genes (13 PCGs, 21 tRNA-coding genes and 2 rRNA-coding genes) and two noncoding regions (OL and d-loop, control region) (Table 4). One of the 13 PCGs (ND6), seven tRNA-coding genes (Gln, Ala, Asn, Cys, Tyr, Ser, Glu), and one noncoding region (OL) are encoded on the L-strand, and the other 28 genes (12 PCGs, 14 tRNA-coding genes, and 2 rRNA-coding genes) and d-loop are encoded on the H-strand. The composition and arrangement of the mitochondrial genes in these four species were the same as those in *T.gangeticus* in the Indian Ocean (MK333401 in Mukundan et al. 2020). To characterize variation among the *Trichiurus* mitogenomes, we analyzed the base composition of *T.gangeticus* in the Indian Ocean (Mukundan et al. 2020). We found the mean AT nucleotide content of the five complete mitogenomes to be similar (55.0% in *T.japonicus*, 55.1% in *T.lepturus*, 54.4% in *T.nanhaiensis*, 54.3% in *T.gangeticus* and 54.6% in *T.brevis*; Table 5). All mitogenomes had high A + T content: 54.3%–55.1% (53.3%–54.1% for PCGs, 54.5%–56.9% for light tRNA genes, 53.4%–54.5% for heavy tRNA genes, 52.3%–52.6% for rRNA genes, and 63.5%–67.1% for d-loop). The overall AT skews in the five entire mitogenomes were 0.06006, 0.04465, 0.05775, 0.04891 and 0.06789, and the overall GC skews were -0.17695, -0.17258, -0.18480, -0.18396 and -0.19633 (Table 5).

**Table 4.** Characteristics of the four newly determined *Trichiurus* mitogenomes.

Gene	Position		Codons		anticodon	Strand	Intergenic nucleotides
	From	To	Start	Stop			
tRNA^Phe^	1/1/1/1	69/69/69/70			GAA	H	0/0/0/-1
12S rRNA	70/70/70/70	1027/1027/1026/1028				H	0/0/0/0
tRNA^Val^	1028/1028/1027/1029	1098/1098/1097/1099			TAC	H	0/0/0/0
16S rRNA	1099/1099/1098/1100	2836/2840/2824/2830				H	0/0/0/0
tRNA^Leu^	2837/2841/2825/2831	2910/2914/2898/2904			TAA	H	0/0/0/0
ND1	2921/2923/2910/2916	3899/3894/3884/3890	TTA	TAA		H	10/9/11/11
tRNA^Ile^	3900/3900/3890/3895	3969/3969/3959/3965			GAT	H	0/5/5/5
tRNA^Gln^	3968/3969/3959/3965	4038/4039/4029/4035			TTG	L	-2/-1/-1/-1
tRNA^Met^	4038/4039/4029/4035	4108/4109/4099/4105			CAT	H	-1/-1/-1/-1
ND2	4110/4111/4101/4107	5156/5157/5147/5153	ATG	TAA		H	1/1/1/1
tRNA^Trp^	5156/5157/5147/5153	5228/5229/5220/5226			TCA	H	-1/-1/-1/-1
tRNA^Ala^	5229/5231/5222/5229	5297/5299/5290/5297			TGC	L	0/1/1/2
tRNA^Asn^	5299/5301/5292/5299	5371/5373/5364/5371			GTT	L	1/1/1/1
O_L_	5374/5376/5367/5374	5403/5405/5396/5403				L	2/2/2/2
tRNA^Cys^	5403/5405/5396/5403	5468/5470/5461/5468			GCA	L	-1/-1/-1/-1
tRNA^Tyr^	5469/5471/5462/5469	5535/5537/5528/5535			GTA	L	0/0/0/0
COI	5537/5539/5530/5537	7087/7089/7080/7087	GTG	TAA		H	1/1/1/1
tRNA^Ser^	7088/7090/7081/7088	7158/7160/7151/7158			TGA	L	0/0/0/0
tRNA^Asp^	7162/7164/7154/7162	7234/7236/7226/7230			GTC	H	3/3/2/3
COII	7236/7240/7229/7235	7926/7930/7919/7925	ATG	TAA		H	1/3/2/4
tRNA^Lys^	7927/7931/7920/7926	7998/8003/7992/7998			TTT	H	0/0/0/0
ATP8	7999/8005/7995/8000	8166/8172/8162/8167	ATG	TAA		H	0/1/2/1
ATP6	8157/8163/8153/8158	8840/8846/8836/8841	ATG	TAA		H	-10/-10/-10/-10
COIII	8840/8846/8836/8841	9625/9631/9621/9626	ATG	TAA		H	-1/-1/-1/-1
tRNA^Gly^	9625/9631/9621/9626	9693/9699/9689/9694			TCC	H	-1/-1/-1/-1
ND3	9694/9700/9690/9695	10044/10050/10040/10045	ATT	TAA		H	0/0/0/0
tRNA^Arg^	10043/10049/10039/10044	10111/10117/10107/10112			TCG	H	-2/-2/-2/-2
ND4L	10112/10118/10108/10113	10408/10414/10404/10409	ATG	TAA		H	0/0/0/0
ND4	10402/10408/10398/10403	11772/11778/11768/11773	ATG	AGA		H	-7/-7/-7/-7
tRNA^His^	11781/11787/11776/11781	11849/11856/11844/11849			GTG	H	8/8/7/8
tRNA^Ser^	11850/11857/11845/11850	11920/11927/11915/11920			GCT	H	0/0/0/0
tRNA^Leu^	11923/11930/11918/11923	11994/12001/11989/11994			TAG	H	2/2/2/2
ND5	11997/12004/11992/11997	13877/13884/13872/13877	ATG	TAA		H	2/2/2/2
ND6	13874/13881/13869/13874	14395/14402/14390/14395	ATG	TAG		L	-4/-4/-4/-4
tRNA^Glu^	14396/14403/14391/14396	14464/14471/14459/14464			TTC	L	0/0/0/0
Cyt b	14469/14476/14464/14469	15609/15616/15604/15609	ATG	TAA		H	4/4/4/4
tRNA^Thr^	15610/15617/15605/15610	15683/15692/15678/15683			TGT	H	0/0/0/0
d-loop	15684/15693/15679/15684	16685/16568/16801/16733				H	

**Table 5.** Nucleotide compositions of *T.japonicus*, *T.lepturus*, *T.nanhaiensis*, *T.brevis*, and *T.gangeticus*.

		Whole genome	Protein-coding genes	Light tRNAs^1^	Heavy tRNAs^2^	2 rRNA	d-loop
AT%	*T.japonicus*	55.0	53.4	56.9	53.4	52.4	66.3
	*T.lepturus*	55.1	54.0	56.4	54.1	52.3	64.4
	*T.nanhaiensis*	54.4	53.3	55.0	54.1	52.5	66.7
	*T.gangeticus* ^3^	54.3	53.5	54.5	54.5	52.3	67.1
	*T.brevis*	54.6	54.1	55.3	53.5	52.6	63.5
AT-skew	*T.japonicus*	0.06006	-0.05230	0.11991	0.11993	0.20156	0.04072
	*T.lepturus*	0.04465	-0.06827	0.09293	0.11745	0.21332	0.00621
	*T.nanhaiensis*	0.05775	-0.05444	0.11080	0.11620	0.21268	-0.01349
	*T.gangeticus* ^3^	0.04891	-0.05679	0.10329	0.10850	0.21337	-0.03428
	*T.brevis*	0.06789	-0.04365	0.11892	0.13515	0.23956	0.02992
GC-skew	*T.japonicus*	-0.17695	-0.29641	-0.05917	-0.20854	-0.16176	-0.11573
	*T.lepturus*	-0.17258	-0.29303	-0.03277	-0.23999	-0.15737	-0.15169
	*T.nanhaiensis*	-0.18480	-0.30426	-0.04978	-0.25498	-0.17127	-0.09910
	*T.gangeticus* ^3^	-0.18396	-0.30589	-0.05000	-0.23819	-0.16780	-0.11246
	*T.brevis*	-0.19633	-0.30975	-0.07410	-0.24391	-0.19198	-0.16164

AT% = [A+T]/[A+T+G+C], AT-skew = [A-T]/[A+T], GC-skew = [G-C]/[G+C]. ^1^ Light tRNAs are those transcribed from the heavy strand mitochondrial DNA, including Phe, Val, Leu, Ile, Met, Trp, Asp, Lys, Gly, Arg, His, Leu, Thr. ^2^ Heavy tRNAs are those transcribed from the light strand, including Gln, Ala, Asn, Cys, Tyr, Ser, Glu. ^3^MK333401 in Mukundan et al. 2020.

**Figure 6.**
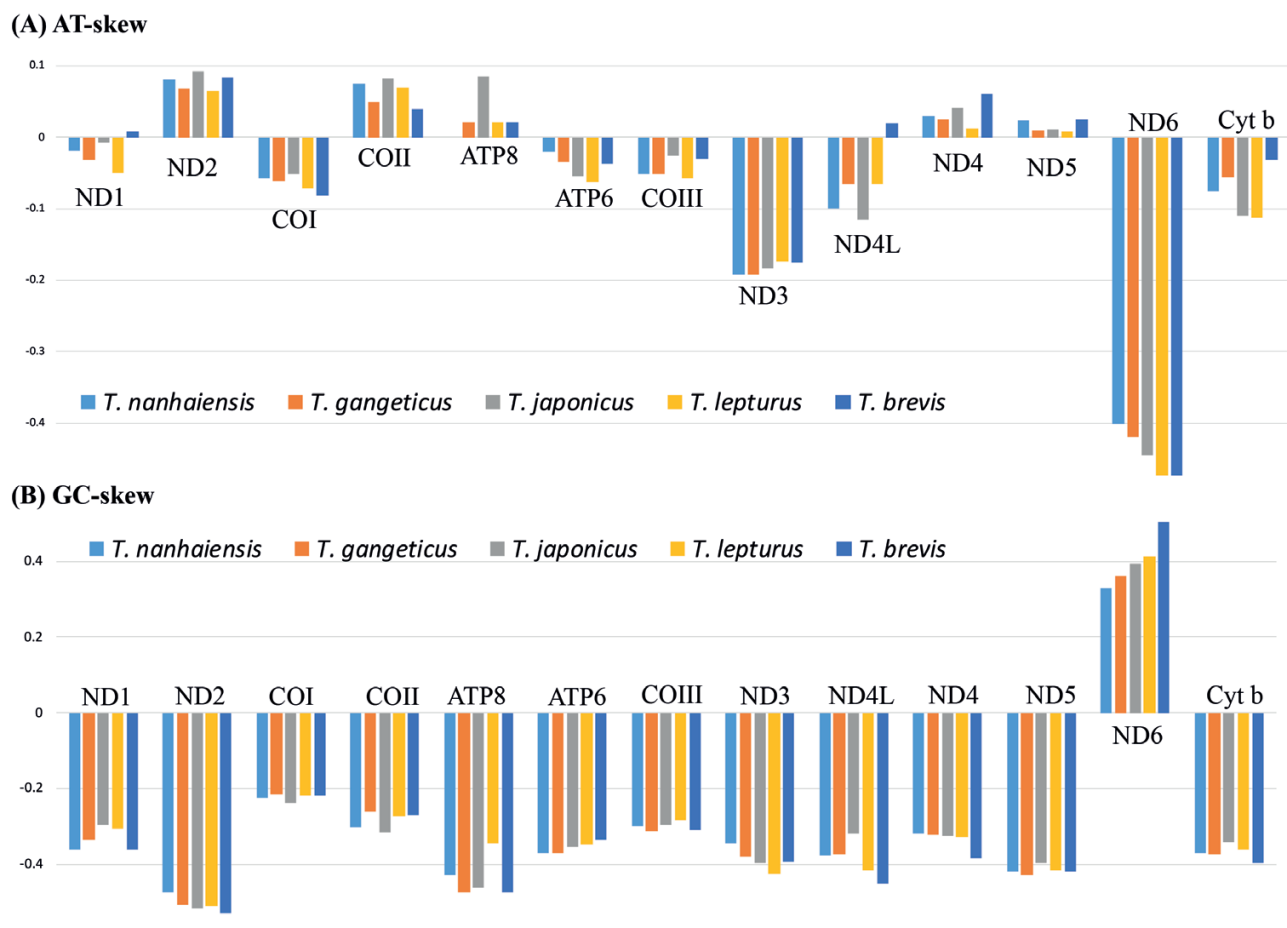
**A** AT-skew in 13genes. **B** GC-skew in in 13 genes.

The total lengths of PCGs in the five *Trichiurus* species ranged from 11.530 to 11538 bp, accounting for 68.47%–69.59% of the entire mitogenome. The mitogenomes could be translated into 3.809–3.810 amino acid-coding codons, excluding stop codons. ND5 and ATP8 were the largest and smallest genes, respectively. The majority of PCGs start with an NTN (ATG/GTG/ATT) start codon and are terminated with the stop codons TAA, TAG, and AGA (Table 4). Most of the AT skew and GC skew values of the PCGs in the five species were negative, indicating that the bases T and C were more plentiful than A and G (Table 5). Moreover, the A + T content and AT skew differed among PCGs (Suppl. material 1: Table S2, Fig. 6). The AT skew values of five genes (ND2, COII, ATP8, ND4 and ND5) were positive, and those of other genes were negative. The GC skew value was positive only for ND6.

**Figure 7.**
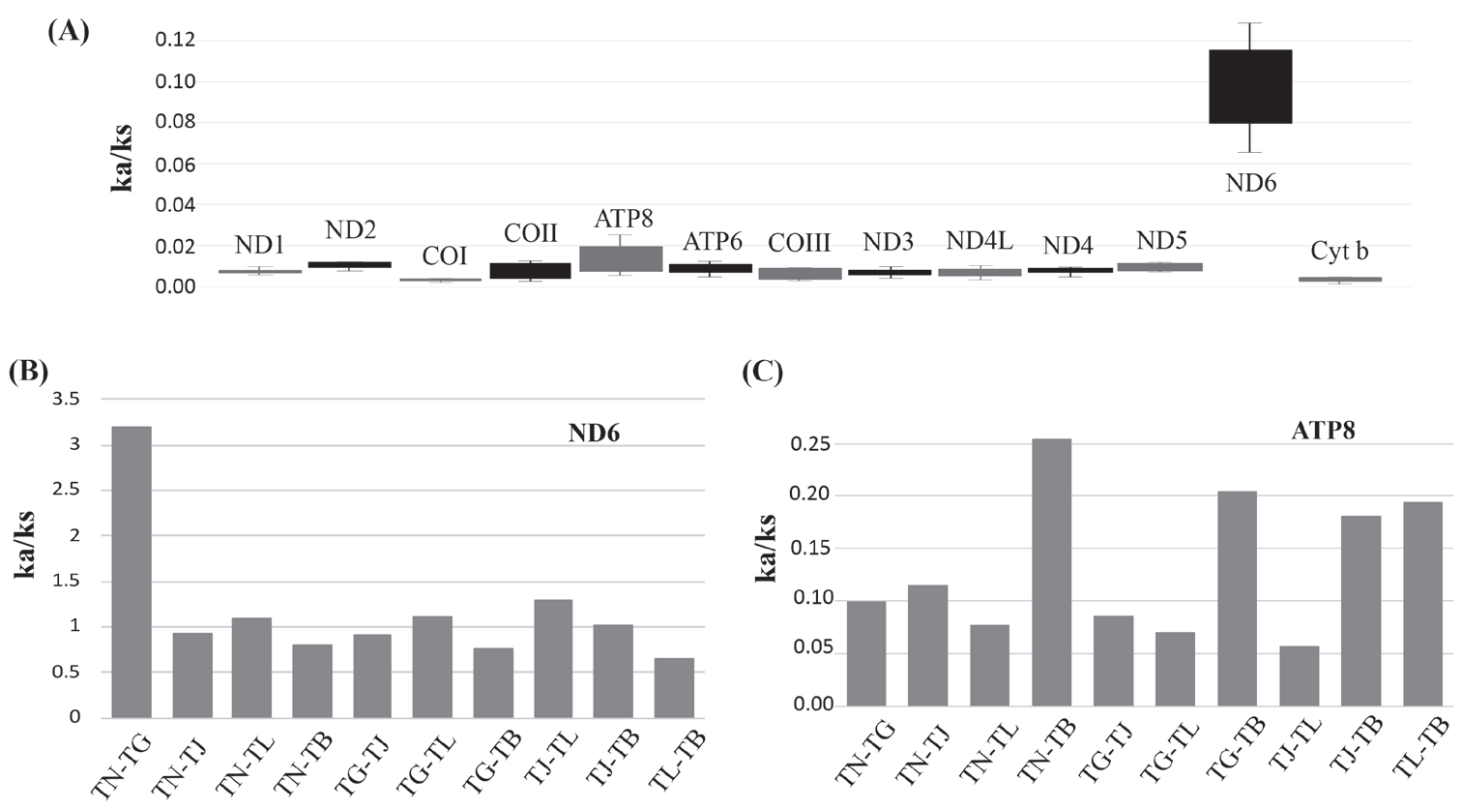
**A** Mean evolutionary rates for each protein coding gene in mitogenomes of five *Trichiurus* species **B** Evolutionary rates of ND6 gene of five *Trichiurus* species. **C** Evolutionary rates of Ka/Ks in ATP8 gene of five *Trichiurus* species. Indicated the rates of non-synonymous substitutions to the rate of synonymous substitutions (ka/ks). *T.japonicus* (TJ), *T.lepturus* (TL), *T.nanhaiensis* (TN), *T.gangeticus* (TG) and *T.brevis* (TB).

**Figure 8.**
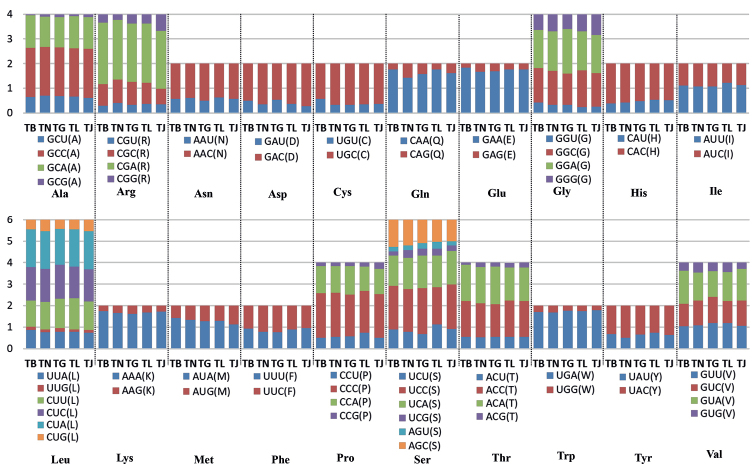
Relative synonymous codon usage (RSCU) of the mitogenomes of the five *Trichiurus* species; the stop codon is not included. *T.japonicus* (TJ), *T.lepturus* (TL), *T.nanhaiensis* (TN), *T.gangeticus* (TG) and *T.brevis* (TB).

**Figure 9.**
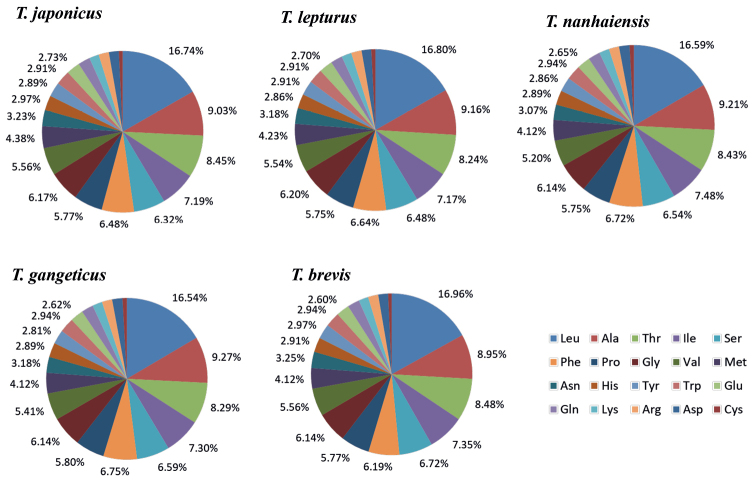
Frequencies of different amino acids in the mitogenomes of the five *Trichiurus* species; the stop codon is not included.

To better understand the role of selection in the evolution of the PCGs, the Ka/Ks value of each PCG was calculated (Fig. 7A). All the PCGs, excluding ND6, showed signatures of purifying selection (Ka/Ks < 1). The ND6 and ATP8 genes had the highest Ka/Ks values (1.18 and 0.13), and the COI and cyt b genes had the lowest Ka/Ks values (0.04). A lower Ka/Ks value indicates less variation in amino acids. For the ND6 gene, the highest Ka/Ks value was observed between *T.nanhaiensis* and *T.gangeticus* (Fig. 7B). For the ATP8 gene, the highest Ka/Ks value was observed between *T.brevis* and other *Trichiurus* species (Fig. 7C). Summaries of the relative synonymous codon usage and number of amino acids in the annotated PCGs are presented in Figs 8, 9 and Suppl. material 1: Table S3. Overall codon usage among the sequenced *Trichiurus* mitogenomes was similar; Leu, Ala, Thr, Ile, and Ser were the five most common amino acids.

The lengths of 16S rRNA genes ranged from 1.725 (*T.gangeticus*) to 1.742 (*T.lepturus*), whereas those of 12S rRNAs ranged from 957 (*T.gangeticus* and *T.nanhaiensis*) to 959 (*T.brevius*). These rRNA genes are located between tRNA^Phe^ and tRNA^Leu^ and are separated by tRNA^Val^. The AT content of the rRNA genes ranged from 52.3% to 52.6% (Table 5). The total lengths of the 21 tRNA genes ranged from 1.483 (*T.japonicus* and *T.brevis*) to 1.487 bp (*T.lepturus*), and individual tRNA genes typically ranged in size from 66 to 76 bp. No sequence similarity to the tRNA^Pro^ gene was observed elsewhere in the mitogenome. The d-loop in *Trichiurus* mitogenomes is located between tRNA^Phe^ and tRNA^Thr^. The A + T content (63.5%–67.1%) of the d-loop was higher than that of the whole genome (54.3%–55.1%), rRNA-coding genes (52.3%–52.6%), and tRNA-coding genes (54.4%–55.2%) (Table 5). Furthermore, compositional analysis revealed that the mitogenome of *T.nanhaiensis* and *T.gangeticus* had a negative AT skew (-0.01349 and -0.03428) in the d-loop.

### ﻿Molecular tool

**Figure 10:**
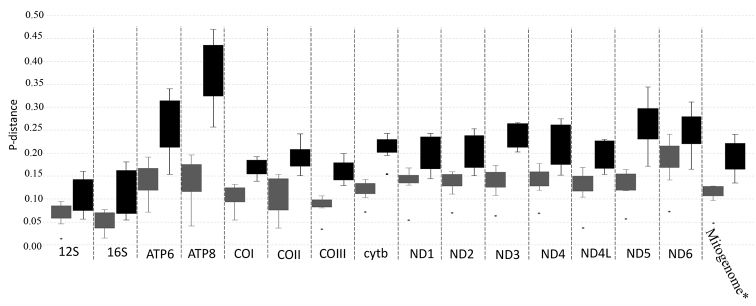
. The mean partwise interspecific (gray) and intergeneric (black) p-distance in each gene.

To determine molecular markers that could be used to examine the phylogeny and identify species, the overall interspecific and intergeneric p-distance was used to describe the evolutionary rate of two rRNA-coding genes, 13 PCGs and the mitogenome, excluding the d-loop region (Fig. 10, Table 6, Suppl. material 1: Table S4). The maximum interspecific p-distance (mean = 0.189, range = 0.073-0.241) was observed for the ND6 gene, and the maximum intergeneric p-distance (mean = 0.369, range = 0.257-0.470) was observed for the ATP8 gene. Among these 16 markers, 9 markers (e.g., 12S rRNA, ATP6 and ND1 genes) displayed overlapping interspecific and intergeneric p-distances (Fig. 10). Among the four genes in the oxidase family, only COII showed overlap between interspecific and intergeneric p-distances. Furthermore, the range of pairwise interspecific p-distances among five *Trichiurus* species based on the 16S rRNA and cyt b genes ranged from 0.015 (between *T.gangeticus* and *T.nanhaiensis*) to 0.077 (between *T.lepturus* and *T.brevis*) and from 0.072 (between *T.gangeticus* and *T.nanhaiensis*) to 0.143 (between *T.nanhaiensis* and *T.brevis*) (Table 6). In addition, our study found that the 16S rRNA genetic distances between *T.brevis* (short-tailed species complex) and other *Trichiurus* species (*T.lepturus* complex or large-head species complex) were not higher than those within the *T.lepturus* complex (Table 6). The results based on cyt b and 16S rRNA differed.

**Table 6.** The p-distance (*10^-2^) between *Trichiurus* species (interspecific) and between genera within Trichiuridae (intergeneric) in each gene and mitogenome (excluding d-loop). *T.japonicus* (TJ), *Trichiuruslepturus* (TL), *Trichiurusnanhaiensis* (TN), *T.gangeticus* (TG), *Trichiurusbrevis* (TB), *Trichiurus* (T), *Benthodesmus* (B), *Aphanopus* (C), *Evoxymetopon* (E), and *Assurger* (A).

	12S	16S	atp6	atp8	COI	COII	COIII	cytb	ND1	ND2	ND3	ND4	ND4L	ND5	ND6	genome
TG/TB	9.4	3.9	17.0	17.3	12.4	14.6	8.3	14.1	13.6	15.0	15.5	17.7	13.8	16.4	23.0	12.8
TG/TJ	6.2	6.2	12.4	11.9	10.5	8.8	8.0	11.4	14.3	14.5	13.2	14.6	12.8	13.0	20.3	11.1
TG/TL	6.5	6.6	14.5	13.1	9.4	7.7	8.4	12.6	14.9	14.8	14.6	14.0	12.1	11.8	20.7	11.1
TG/TN	1.4	1.5	7.2	4.2	5.4	3.6	3.4	7.2	5.4	7.0	6.3	6.9	3.7	5.7	7.3	4.8
TB/TJ	7.6	7.4	14.8	15.5	13.2	13.9	10.3	13.2	16.8	14.9	16.5	15.7	16.8	15.3	17.8	12.8
TB/TL	8.3	7.7	19.2	19.6	12.8	15.3	10.7	13.2	15.7	15.6	15.1	15.2	14.8	14.1	19.7	12.9
TB/TN	9.4	2.8	16.7	18.5	11.5	14.3	9.5	14.3	13.0	13.5	17.4	16.8	15.5	16.1	24.1	12.4
TJ/TL	4.6	4.9	12.1	10.7	9.4	9.0	9.7	10.3	15.1	11.1	10.8	12.0	10.4	12.2	14.2	9.7
TJ/TN	6.4	6.4	11.1	13.1	9.7	8.3	8.9	11.9	14.3	16.0	14.5	13.9	14.8	13.7	21.1	11.3
TL/TN	6.5	6.9	14.5	15.5	9.6	7.5	9.4	12.5	14.7	15.3	15.7	13.1	13.5	11.9	20.9	11.2
T/B	16.0	18.1	34.0	47.0	19.	24.2	19.9	22.6	23.3	25.3	26.6	26.6	22.6	34.4	31.1	24.1
T/C	15.2	17.0	31.4	43.3	18.9	22.1	18.9	24.3	23.5	23.6	26.5	25.3	23.0	31.7	30.0	22.9
T/E	14.0	15.4	30.7	40.6	17.5	20.4	16.3	20.4	24.3	23.8	25.2	26.0	23.0	28.8	27.3	21.5
T/A	14.0	16.0	31.5	44.3	18.3	20.4	17.6	21.2	23.5	24.0	23.1	27.5	22.3	29.1	26.9	21.9
B/C	7.6	7.0	21.6	26.3	13.9	17.1	12.9	19.5	14.4	15.2	21.5	15.2	15.4	28.7	19.3	16.1
B/E	9.7	9.1	25.1	35.7	17.0	19.9	16.8	20.9	16.9	19.6	22.6	17.7	17.1	26.4	24.3	18.2
B/A	11.6	10.1	24.9	37.4	16.4	20.3	15.9	20.7	17.5	18.5	26.4	20.2	19.1	27.9	25.5	18.9
C/E	7.2	6.5	20.2	34.5	16.4	18.2	14.4	22.2	16.7	17.7	21.8	17.0	18.1	22.4	23.0	16.7
C/A	7.7	7.3	21.8	34.5	15.9	17.2	15.3	24.2	17.5	17.4	20.6	19.5	18.8	23.3	23.4	17.0
E/A	5.6	5.4	15.4	25.7	14.2	15.1	13.5	15.4	16.2	15.1	20.3	17.9	15.4	17.2	16.5	13.5

## ﻿Discussion

### ﻿Mitogenomic features of *Trichiurus* species

The mitogenomes of *Trichiurus* species encode 36 typical mitochondrial genes (13 protein-coding, 2 ribosomal RNA-coding genes, and 21 transfer RNA-coding genes) and two typical noncoding control regions, the d-loop and origin of the light strand (OL) (Table 4). Overall, the mitogenomes of four *Trichiurus* species in the present study have a gene order and composition similar to other *Trichiurus* mitogenomes in previous studies (e.g., Liu and Cui 2009; Liu et al. 2013; Zheng et al. 2019; Mukundan et al. 2020). The gene order of the mitogenomes in Trichiuridae was similar to that in most teleosts, although different types of gene rearrangements were observed within Pleuronectiformes (Gong et al. 2015) and Stomiiformes (Arrondo et al. 2020).

In addition, our study found that the tRNA^Pro^ gene was absent in the *Trichiurus* mitogenomes. Previous studies of the complete mitogenomes of *Trichiurus* species have also obtained similar findings (e.g., Liu and Cui 2009; Liu et al. 2013; Zheng et al. 2019; Mukundan et al. 2020). Our data indicate that this event only occurred in the genus *Trichiurus* and not in other teleosts. Adams and Palmer (2003) proposed that the mitochondrial gene content is highly variable across eukaryotes. However, most previous studies have been conducted on plants (Adams et al. 2001; Adams and Palmer 2003). In addition, the loss of genes in vertebrate mitogenomes is rare. In teleosts, loss of the ND6 gene was observed only in Antarctic fish mitogenomes (Papetti et al. 2007), and no cases of tRNA gene loss were observed.

### ﻿Molecular tool assessment

Because the systematics of many species remain unresolved, many studies have employed molecular, phylogenetics and DNA barcoding approaches (e.g., Hebert et al. 2003; Hsu et al. 2009; Han et al. 2019). Among all molecular markers, the mtDNA COI and cyt b genes have been the most frequently used (Yang et al. 2016; Han et al. 2019; Hsu et al. 2020; Ju et al. 2021). In addition, some studies have used mtDNA rRNA (12S and 16S) sequences to resolve phylogenetic relationships and taxonomy (Byrne et al. 2010; Herler et al. 2013; Zheng et al. 2016). However, the results of pairwise p-distances based on 16S rRNA and cyt b genes differed in this study (Table 6). These results, coupled with the results of the Ka/Ks analyses (Fig. 7), suggested that the evolutionary rates of these genes differed. Our study indicated that overlap between the interspecific and intergeneric distances might affect phylogenetic reconstruction and molecular species identification. For example, the intergeneric 12S rRNA p-distance between *Evoxymetopon* and *Assurger* was 0.056, which is smaller than the interspecific 12S rRNA p-distance of *Trichiurus* (Table 6, Fig. 10). Thus, some genes that were used to resolve the phylogeny and identify species should be evaluated. Actually, this question has been intensively discussed, and has been applied to mitochondrial genes (i.e., Zardoya and Meyer 1996; Miya and Nishida 2000). However, some studies ignored this question (our observations).

Moreover, variation in the Ka/Ks values was greater for ATP8 than for other genes (Fig. 7), and the length of ATP8 (approximately 168 bp) was shorter. Thus, our study suggested that only COI, COIII, cyt b, ND5 and mitogenome (excluding d-loop) sequences could be used to identify *Trichiurus* species and examine the phylogeny of Trichiuridae. However, these genes may also display a limited ability to identify complex evolutionary relationships in many fishes (Mirande 2018). For example, the Ka/Ks values of the COI and cyt b genes were the lowest (0.04). Lower Ka/Ks values indicate less variation in amino acids (Brookfiel 2000; Li et al. 2020; Sun et al. 2021). Therefore, an increasing number of studies have used complete mitogenome data to resolve animal phylogenies and identify species because they provide more information (Ajene et al. 2020; González-Castellano et al. 2020; Irisarri et al. 2020); the results of our study support this hypothesis.

### ﻿Systematics of *Trichiurus*

The taxonomy of the genus *Trichiurus* remains unresolved because of the high degree of morphological similarity within the genus in terms of bodily appearance and silvery coloration. Our study also showed that identifying *Trichiurus* species by morphological characters is very difficult (Fig. 5, Table 3). Phylogenetic analyses based on the complete mitogenome (Fig. 3) and COI gene (Fig. 4) showed that *T.haumela* was clustered with *T.japonicus*. Moreover, *T.japonicus* is synonymous with *T.lepturus* in FishBase (Froese and Pauly 2021), but the present results (Figs 3, 4; Table 2) indicated that *T.haumela* is synonymous with *T.japonicus* and that *T.japonicus* is a valid species (Hsu et al. 2009; Fricke et al. 2021). Moreover, the systematic position of *T.brevis* is still not resolved in this study because we did not analyze other species of *Trichiurusrusselli* complex, and did not provide enough information.

In addition, the results suggested that specimens in the Indian Ocean (MK333401 in Mukundan et al. 2020) are not “*T.lepturus*” (Figs 3, 4; Table 2). In the phylogenetic tree based on COI (Fig. 4), MK333401 was grouped with other specimens in the Indian Ocean as lineage E. Within lineage E, most specimens were identified as “*T.lepturus*”, and only MK340737 in Bangladesh was identified as *T.gangeticus*. According to these results, members of lineage E could not be identified as *T.lepturus*; our data suggest that they should be recognized as *T.gangeticus*. Similarly, within lineage B, some specimens were identified as *Trichiurus* sp. (Isari et al. 2017), and some specimens were identified as *T.auriga*. We thus recognized lineage B as *T.auriga* (Fig. 4). Accordingly, our study suggests that the *Trichiurus* specimens in the Indian Ocean are not *T.lepturus* calls into question many previous studies (e.g., Chakraborty et al. 2006a; Jahromi et al. 2016; Mukundan et al. 2020).

Chakraborty et al. (2006a) and Chakraborty and Iwatsuki (2006) found that *T.lepturus* in Indo-Pacific differed from that in Atlantic using 16S rRNA sequences. However, Hsu et al. (2009) identified the specimens of *T.lepturus* in the Indo-Pacific in Chakraborty et al. (2006a) and Chakraborty and Iwatsuki (2006) as “*Trichiurus* sp. 2” (synonym of *T.nanhaiensis*). Jahromi et al. (2016) examined the phylogenetic relationship of *T.lepturus* from the Persian Gulf using 16S rRNA sequences, and suggested homogeneity between Persian Gulf and the other Indo-Pacific individuals. However, Lin et al. (2021) found that the specimens in Jahromi et al. (2016) were identified as *T.japonicus*, *T.lepturus*, and *T.nanhaiensis* and the specimens in the Persian Gulf was nested with *T.nanhaiensis* using 16S rRNA sequences. Besides, Lin et al. (2021) found *T.nanhaiensis* could be divide as two groups, Indo-Pacific and West Indian. The results of our study indicate that *T.gangeticus* was more similar to *T.nanhaiensis* (Fig. 4, Table 2). Thus, our study considers these two groups might be *T.nanhaiensis* and *T.gangeticus*, although we did not collect the COI data of *T.nanhaiensis* in the east Indian Ocean. In addition, our study found some specimens from the Gulf of Oman referred to in Lin et al. (2021) were in fact *T.lepturus*. However, *T.lepturus* had the highest intraspecific diversity (Table 2). Thus, our study suggests that systematics of *T.lepturus* species complex and *T.lepturus* both need to be reviewed.

Bingpeng et al. (2018) used the COI gene to identify fish at the species level in the Taiwan Strait and proposed that the average p-distances within species, genera, families, orders, and classes were 0.0021, 0.0650, 0.2370, and 0.2560, respectively. Our study revealed that the range of COI interspecific distances in *Trichiurus* ranged from 0.0435 to 0.1600, and the intraspecific distance within lineage C (*T.lepturus*) was 0.0333 (Table 2, Fig. 4). These results suggest that there were cryptic species within lineage C. Lineage C could be divided into three sublineages C1–C3 (Fig. 4). Lineage C1 was distributed in the West Pacific Ocean; lineage C2 was distributed in the Northwest Atlantic Ocean; and lineage C3 was distributed in the East Pacific and Southwest Atlantic oceans. The range of the pairwise genetic distances ranged from 0.0308 to 0.0529. Thus, these three sublineages should be recognized as three different species. Within lineage C3, most specimens were identified as *T.lepturus*, but some specimens (MF957079-MF957087) were identified as *T.nitens*. *Trichiurusnitens* was described in 1899, and it is distributed in the eastern Pacific, from California south to Peru. Nakamura and Parin (1993) considered it synonymous with *T.lepturus*, but some researchers have suggested that it is the real *T.nitens* (Eschmeyer and Herald 1983; Burhanuddin and Parin 2008; Robertson et al. 2017). In addition, *T.margarites* is considered a valid species in FishBase (Froese and Pauly 2021) and ECoF (Fricke et al. 2021). *Trichiurusmargarites* is distributed in the South China Sea (Li 1992; Fricke et al. 2021), but this species has not yet been detected along Chinese coastal waters. However, it is possible that the lineage C1 is *T.margarites* (Fig. 4). Thus, our study suggested that the systematics within lineage C require careful evaluation. In future studies, a careful morphological comparative work within lineage C is needed.

### ﻿Morphological analyses

Tzeng et al. (2007) analyzed the morphometry from *T.japonicus* and *T.lepturus*, and found that it exhibited high intraspecific variations. Our study also found the same (Fig. 5, Table 3). However, although Tzeng et al. (2007) found a decisive specific gap of non-overlapping scattering using discriminant function analysis, they did not provide a reference key to identify the *Trichiurus* species because it is very difficult. Lee et al. (1977) proposed that *T.japonicus* and *T.lepturus* can been distinguished based on the external morphology of various body ratios. Thus, our study calculated some body ratios (Table 3, Fig. 5) and only found that the ratio between length and depth of head can been used to distinguish *T.lepturus* and other species (Fig. 5D). In *T.lepturus*, the ratio between distance of head length [D(i,l)] and distance of head depth [D(d,o)] was larger than 2.5. Our study also did not find a reference key to distinguish these three species within *T.lepturus* complex; we used the complex indexes to distinguish them. *Trichiurusjaponicus* has a longer body and tail (Fig. 5A, B); *T.lepturus* has a shorter tail, longer head, and bigger eye (Fig. 5A, C, D); and *T.nanhaiensis* has a wider tail, smaller eye, and shorter head (Fig. 5B, C, D).

## ﻿Conclusions

Accurate species identification is important for fishery purposes. The current study represents the first comparative mitogenomic and phylogenetic analysis within *Trichiurus* and provides new insight into the mitogenomic features and evolution of fishes. Our study suggested that (1) it is difficult to identify species of *T.lepturus* complex by morphology; (2) *T.japonicus* is a valid species; and (3) the specimens in Indian Ocean are neither *T.lepturus* nor *T.nanhaiensis*. Furthermore, Shih et al. (2011) proposed that the von Bertalanffy growth model of three *Trichiurus* species in Taiwanese waters differed. Thus, accurate species identification of *Trichiurus* species for resource management is very important. Our study identified four *Trichiurus* species along the China Sea coasts. The historical records of their distribution were *T.japonicus* in the Northwestern Pacific, China, and Taiwan to Japan, *T.lepturus* in tropical and warm temperate seas, (including Gulf of Mexico, Caribbean Sea, Mediterranean Sea, Sea of Marmara, Red Sea, Persian Gulf), *T.nanhaiensis* in the West Pacific, and *T.brevis* in the South China Sea (Fricke et al. 2021). Thus, our team wants to sample more specimens in other regions. We hope that our current results can provide more information on the systematics and diversity of *Trichiurus*. Future studies should collect more specimens in the Indian Ocean to re-examine the systematics of *Trichiurus* by mitogenomic, nuclear gene, and morphological data. The results of this study also have implications for the resource management of *Trichiurus* species.

## Supplementary materials

Supplementary material 1 Table S1–S4, Figure S1, S2 Data type docx file Explanation note **Table S1.** Sample sizes of the genus *Trichiurus* in 18 sampling locations in Figure 1A. **Table S2.** Nucleotide compositions of *T.japonicus* (TJ), *T.lepturus* (TL), *T.nanhaiensis* (TN), *T.gangeticus* (TG) and *T.brevis* (TB). **Table S3.** Total number and frequency of the codons in mitogenomes. **Table S4.** The maximum (max.), mean and minimum (min.) p-distances between Trichiurus species (interspecific) and between genera within Trichiuridae (intergeneric) in each gene and mitogenome. **Figure S1.** Frontal view of the heads of preserved specimens of *T.lepturus* species complex. A *T.nanhaiensis*, 83.4 cm TL, ZBL 000440, Zhanjiang; B *T.japonicus*, 85.6 cm TL, ZJ 1902, Zhanjiang; C *T.lepturus*, 76.3 cm TL, ZJ 1906, Zhanjiang. **Figure S2.** The boxplot analyses in *T.japonicus* (blue), *T.lepturus* (orange) and *T.nanhaiensis* (grey). The landmarks are illustrated in Fig. 2. https://binary.pensoft.net/file/638355This dataset is made available under the Open Database License (http://opendatacommons.org/licenses/odbl/1.0/). The Open Database License (ODbL) is a license agreement intended to allow users to freely share, modify, and use this Dataset while maintaining this same freedom for others, provided that the original source and author(s) are credited.Mu-Rong Yi, Kui-Ching Hsu, Sui Gu, Xiong-Bo He, Zhi-Sen Luo, Hung-Du Lin, Yun-Rong Yan
