# Supplementary material for: ﻿Complete mitogenomes of four Trichiurus species: A taxonomic review of the T.lepturus species complex
Source: Zookeys. 2022 Jan 26;1084:1–26. doi: 10.3897/zookeys.1084.71576 (PMC8810657; doi:10.3897/zookeys.1084.71576)
Supplement: Supplementary material 1 — Table S1–S4, Figure S1, S2 [file zookeys-1084-001-s001.docx]

| **Table S1.** Sample sizes of the genus *Trichiurus* in 18 sampling locations in Figure 1A. | | | | |
| --- | --- | --- | --- | --- |
| Locality (Abbr.) | *T. japonicus* | *T. lepturus* | *T. nanhaiensis* | *T. brevis* |
| 1.Dandong (DD) | 70 | 0 | 0 | 0 |
| 2.Dalian (DL) | 99 | 0 | 0 | 0 |
| 3.Weihai (WH) | 84 | 0 | 0 | 0 |
| 4.Qingdao (QD) | 99 | 0 | 0 | 0 |
| 5.Lianyungang (LY) | 38 | 0 | 0 | 0 |
| 6.Nantong (NT) | 86 | 0 | 0 | 0 |
| 7.Zhoushan (ZS) | 93 | 0 | 0 | 0 |
| 8.Wenzhou (WZ) | 71 | 0 | 0 | 0 |
| 9.Ningde (ND) | 60 | 0 | 0 | 0 |
| 10.Quanzhou (QZ) | 89 | 0 | 0 | 0 |
| 11.Shantou (ST) | 5 | 0 | 64 | 12 |
| 12.Shenzhen (SZ) | 0 | 0 | 52 | 55 |
| 13.Yangjiang (YJ) | 43 | 0 | 0 | 12 |
| 14.Zhanjiang (ZJ) | 18 | 7 | 4 | 0 |
| 15.Beihai (BH) | 65 | 0 | 0 | 6 |
| 16.Wenchang (WC) | 9 | 0 | 0 | 81 |
| 17.Sanya (SY) | 29 | 0 | 0 | 40 |
| 18. Taidong (TD) | 0 | 20 | 0 | 0 |
| **Total** | **958** | **27** | **120** | **206** |

| **Table S2.** Nucleotide compositions of *T. japonicus* (TJ), *T. lepturus* (TL), *T. nanhaiensis* (TN), *T.* *gangeticus* (TG), and *T. brevis* (TB). | | | | | | | | | | | | | | | | | |
| --- | --- | --- | --- | --- | --- | --- | --- | --- | --- | --- | --- | --- | --- | --- | --- | --- | --- |
|  | AT% | | | | |  | AT-skew | | | | |  | GC-skew | | | | |
|  | TJ | TL | TN | TG | TB |  | TJ | TL | TN | TG | TB |  | TJ | TL | TN | TG | TB |
| tRNA^Phe^ | 56.5 | 53.6 | 49.2 | 47.8 | 52.9 |  | 0.384 | 0.300 | 0.354 | 0.393 | 0.244 |  | 0.000 | 0.063 | -0.030 | -0.056 | 0.091 |
| 12S rRNA | 52.4 | 51.5 | 52.6 | 52.2 | 52.5 |  | 0.172 | 0.208 | 0.179 | 0.184 | 0.246 |  | -0.118 | -0.126 | -0.131 | -0.126 | -0.173 |
| tRNA^Val^ | 66.2 | 63.4 | 56.4 | 56.4 | 60.6 |  | 0.063 | 0.066 | 0.100 | 0.099 | 0.069 |  | -0.083 | 0.000 | -0.032 | -0.032 | -0.071 |
| 16S rRNA | 52.3 | 53.0 | 52.3 | 52.3 | 52.7 |  | 0.231 | 0.219 | 0.247 | 0.243 | 0.233 |  | -0.206 | -0.189 | -0.212 | -0.210 | -0.211 |
| tRNA^Leu^ | 54.1 | 54.1 | 50.0 | 50.0 | 50.0 |  | 0.050 | 0.050 | 0.188 | 0.188 | 0.188 |  | -0.059 | -0.059 | -0.136 | -0.136 | -0.136 |
| ND1 | 54.4 | 52.0 | 52.8 | 53.1 | 55.1 |  | -0.007 | -0.050 | -0.019 | -0.032 | 0.009 |  | -0.294 | -0.306 | -0.360 | -0.335 | -0.36 |
| tRNA^Ile^ | 47.1 | 48.6 | 45.7 | 42.9 | 49.3 |  | 0.091 | 0.058 | 0.063 | 0.068 | 0.144 |  | -0.027 | 0.111 | 0.053 | 0.051 | 0.000 |
| tRNA^Gln^ | 57.7 | 57.8 | 60.6 | 60.6 | 56.3 |  | 0.172 | 0.121 | 0.116 | 0.069 | 0.201 |  | -0.201 | -0.267 | -0.428 | -0.357 | -0.291 |
| tRNA^Met^ | 53.6 | 53.6 | 52.2 | 52.2 | 52.2 |  | 0.000 | -0.052 | 0.027 | 0.027 | 0.027 |  | -0.092 | -0.092 | -0.119 | -0.119 | -0.177 |
| ND2 | 52.9 | 53.3 | 52.2 | 52.8 | 52.6 |  | 0.093 | 0.066 | 0.080 | 0.068 | 0.084 |  | -0.516 | -0.507 | -0.473 | -0.505 | -0.528 |
| tRNA^Trp^ | 45.2 | 48.0 | 44.6 | 43.3 | 44.6 |  | 0.274 | 0.200 | 0.090 | 0.062 | 0.090 |  | 0.051 | 0.000 | -0.025 | 0.000 | -0.025 |
| tRNA^Ala^ | 60.8 | 59.4 | 60.8 | 60.8 | 60.9 |  | 0.191 | 0.172 | 0.191 | 0.191 | 0.238 |  | -0.258 | -0.215 | -0.258 | -0.258 | -0.258 |
| tRNA^Asn^ | 52.0 | 52.0 | 50.7 | 52.0 | 50.7 |  | 0.212 | 0.212 | 0.243 | 0.212 | 0.243 |  | -0.200 | -0.257 | -0.168 | -0.144 | -0.168 |
| O_L_ | 53.4 | 53.4 | 53.3 | 51.6 | 53.3 |  | 0.000 | 0.000 | 0.126 | 0.000 | 0.126 |  | -0.143 | -0.143 | -0.143 | -0.198 | -0.143 |
| tRNA^Cys^ | 56.1 | 57.6 | 56.1 | 56.1 | 56.1 |  | -0.027 | -0.052 | -0.080 | -0.027 | -0.080 |  | -0.241 | -0.285 | -0.309 | -0.309 | -0.309 |
| tRNA^Tyr^ | 40.3 | 44.8 | 44.8 | 44.8 | 44.8 |  | 0.112 | 0.134 | 0.134 | 0.134 | 0.134 |  | -0.149 | -0.188 | -0.188 | -0.188 | -0.188 |
| COI | 53.3 | 53.0 | 52.4 | 52.4 | 53.3 |  | -0.051 | -0.072 | -0.057 | -0.061 | -0.081 |  | -0.238 | -0.217 | -0.225 | -0.214 | -0.219 |
| tRNA^Ser^ | 49.3 | 50.7 | 47.9 | 49.3 | 50.8 |  | 0.030 | 0.057 | 0.061 | 0.030 | 0.000 |  | -0.168 | -0.201 | -0.190 | -0.168 | -0.201 |
| tRNA^Asp^ | 64.4 | 61.6 | 61.6 | 63.0 | 60.9 |  | -0.022 | -0.023 | 0.023 | 0.000 | -0.048 |  | -0.079 | 0.070 | 0.070 | 0.038 | -0.038 |
| COII | 55.6 | 55.5 | 54.7 | 56.6 | 56.0 |  | 0.083 | 0.070 | 0.075 | 0.049 | 0.039 |  | -0.315 | -0.274 | -0.302 | -0.260 | -0.268 |
|  |  |  |  |  |  |  |  |  |  |  |  |  |  |  |  |  |  |
| **Table S2.** Continued. | | | | | | | | | | | | | | | | | |
|  | AT% | | | | |  | AT-skew | | | | |  | GC-skew | | | | |
|  | TJ | TL | TN | TG | TB |  | TJ | TL | TN | TG | TB |  | TJ | TL | TN | TG | TB |
| tRNA^Lys^ | 58.3 | 57.5 | 58.9 | 58.9 | 57.5 |  | 0.142 | 0.190 | 0.117 | 0.117 | 0.190 |  | -0.266 | -0.289 | -0.202 | -0.202 | -0.289 |
| ATP8 | 56.0 | 58.4 | 58.4 | 57.2 | 54.8 |  | 0.086 | 0.021 | 0.000 | 0.021 | 0.022 |  | -0.459 | -0.343 | -0.429 | -0.472 | -0.473 |
| ATP6 | 54.8 | 56.1 | 54.1 | 54.5 | 53.8 |  | -0.055 | -0.062 | -0.020 | -0.035 | -0.037 |  | -0.354 | -0.347 | -0.369 | -0.370 | -0.333 |
| COIII | 50.7 | 52.8 | 50.6 | 51.0 | 52.8 |  | -0.026 | -0.057 | -0.051 | -0.051 | -0.030 |  | -0.294 | -0.284 | -0.300 | -0.310 | -0.309 |
| tRNA^Gly^ | 68.1 | 68.1 | 69.5 | 69.5 | 66.7 |  | 0.063 | 0.063 | 0.042 | 0.042 | 0.043 |  | -0.091 | -0.091 | -0.046 | -0.145 | -0.129 |
| ND3 | 51.0 | 52.5 | 52.2 | 51.0 | 53.8 |  | -0.184 | -0.173 | -0.192 | -0.192 | -0.175 |  | -0.396 | -0.424 | -0.344 | -0.380 | -0.394 |
| tRNA^Arg^ | 65.2 | 66.7 | 65.2 | 65.2 | 65.2 |  | 0.067 | 0.043 | 0.110 | 0.110 | 0.067 |  | 0.000 | -0.045 | 0.000 | 0.000 | 0.000 |
| ND4L | 49.5 | 51.8 | 50.2 | 50.5 | 50.8 |  | -0.115 | -0.066 | -0.100 | -0.065 | 0.020 |  | -0.319 | -0.414 | -0.378 | -0.374 | -0.451 |
| ND4 | 53.8 | 55.3 | 54.6 | 54.4 | 54.1 |  | 0.041 | 0.013 | 0.029 | 0.026 | 0.061 |  | -0.325 | -0.329 | -0.319 | -0.320 | -0.381 |
| tRNA^His^ | 65.2 | 62.9 | 66.6 | 68.1 | 65.2 |  | 0.110 | 0.046 | 0.174 | 0.148 | 0.156 |  | 0.167 | 0.231 | 0.045 | 0.091 | 0.084 |
| tRNA^Ser^ | 47.9 | 50.7 | 49.3 | 46.5 | 47.9 |  | 0.353 | 0.333 | 0.201 | 0.153 | 0.236 |  | -0.190 | -0.144 | -0.112 | 0.000 | -0.083 |
| tRNA^Leu^ | 57.0 | 51.4 | 58.3 | 55.6 | 55.6 |  | 0.074 | 0.136 | 0.094 | 0.101 | 0.101 |  | -0.033 | -0.086 | 0.000 | 0.000 | -0.063 |
| ND5 | 53.6 | 55.5 | 53.5 | 53.9 | 55.4 |  | 0.011 | 0.009 | 0.024 | 0.009 | 0.025 |  | -0.397 | -0.416 | -0.419 | -0.427 | -0.417 |
| ND6 | 54.4 | 51.7 | 52.5 | 54.8 | 56.2 |  | -0.445 | -0.474 | -0.402 | -0.420 | -0.473 |  | 0.395 | 0.412 | 0.331 | 0.363 | 0.503 |
| tRNA^Glu^ | 57.9 | 56.5 | 57.9 | 57.9 | 55.0 |  | 0.150 | 0.179 | 0.150 | 0.150 | 0.211 |  | -0.243 | -0.267 | -0.243 | -0.243 | -0.292 |
| Cyt b | 54.4 | 54.5 | 54.1 | 53.8 | 54.7 |  | -0.110 | -0.112 | -0.076 | -0.056 | -0.031 |  | -0.342 | -0.361 | -0.368 | -0.372 | -0.395 |
| tRNA^Thr^ | 47.3 | 48.7 | 41.9 | 43.3 | 45.9 |  | 0.027 | -0.080 | -0.031 | -0.062 | 0.059 |  | -0.127 | -0.127 | -0.164 | -0.190 | -0.200 |
| d-loop | 66.3 | 64.4 | 66.7 | 67.1 | 63.5 |  | 0.041 | 0.006 | -0.013 | -0.034 | 0.030 |  | -0.116 | -0.152 | -0.099 | -0.112 | -0.162 |

| **Table S3.** Total number and frequency of the codons in mitogenomes. | | | | | | | | |
| --- | --- | --- | --- | --- | --- | --- | --- | --- |
| *T. japonicus* | *T. lepturus* | *T. nanhaiensis* | *T. gangeticus* | *T. brevis* | *B. tenuis* | *A. carbo* | *E. poeyi* | *A. anzac* |
| 3811 | 3809 | 3810 | 3810 | 3810 | 3804 | 3803 | 3803 | 3803 |
| Leu (16.74%) | Leu (16.80%) | Leu (16.59%) | Leu (16.54%) | Leu (16.96%) | Leu (16.54%) | Leu (16.96%) | Leu (16.59%) | Leu (16.74%) |
| Ala (9.03%) | Ala (9.16%) | Ala (9.21%) | Ala (9.27%) | Ala (8.95%) | Ala (9.27%) | Ala (8.95%) | Ala (9.21%) | Ala (9.03%) |
| Thr (8.45%) | Thr (8.24%) | Thr (8.43%) | Thr (8.29%) | Thr (8.84%) | Thr (8.29%) | Thr (8.48%) | Thr (8.43%) | Thr (8.45%) |
| Ile (7.19%) | Ile (7.17%) | Ile (7.48%) | Ile (7.30%) | Ile (7.35%) | Ile (7.30%) | Ile (7.35%) | Ile (7.48%) | Ile (7.19%) |
| Phe (6.48%) | Phe (6.64%) | Phe (6.72%) | Phe (6.75%) | Ser (6.72%) | Ser (6.75%) | Ser (6.72%) | Ser (6.72%) | Ser (6.48%) |
| Ser (6.32%) | Ser (6.48%) | Ser (6.54%) | Ser (6.59%) | Phe (6.19%) | Phe (6.59%) | Phe (6.19%) | Phe (6.54%) | Phe (6.32%) |
| Gly (6.17%) | Gly (6.20%) | Gly (6.14%) | Gly (6.14%) | Gly (6.14%) | Pro (6.14%) | Pro (6.14%) | Pro (6.14%) | Pro (6.17%) |
| Pro (5.77%) | Pro (5.75%) | Pro (5.75%) | Pro (5.80%) | Pro (5.77%) | Gly (5.80%) | Gly (5.77%) | Gly (5.75%) | Gly (5.77%) |
| Val (5.56%) | Val (5.54%) | Val (5.20%) | Val (5.41%) | Val (5.56%) | Val (5.41%) | Val (5.56%) | Val (5.20%) | Val (5.56%) |
| Met (4.38%) | Met (4.23%) | Met (4.12%) | Met (4.12%) | Met (4.12%) | Met (4.12%) | Met (4.12%) | Met (4.12%) | Met (4.38%) |
| Asn (3.23%) | Asn (3.18%) | Asn (3.07%) | Asn (3.18%) | Asn (3.25%) | Asn (3.18%) | Asn (3.25%) | Asn (3.07%) | Asn (3.23%) |
| His (2.97%) | Tyr (2.91%) | Trp (2.94%) | Trp (2.94%) | Tyr (2.97%) | His (2.94%) | His (2.97%) | His (2.94%) | His (2.97%) |
| Trp (2.91%) | Trp (2.91%) | His (2.89%) | His (2.89%) | Trp (2.94%) | Tyr (2.89%) | Tyr (2.94%) | Tyr (2.89%) | Tyr (2.91%) |
| Tyr (2.89%) | His (2.86%) | Tyr (2.86%) | Tyr (2.81%) | His (2.91%) | Trp (2.81%) | Trp (2.91%) | Trp (2.86%) | Trp (2.89%) |
| Glu (2.73%) | Glu (2.70%) | Glu (2.65%) | Glu (2.62%) | Glu (2.60%) | Glu (2.62%) | Glu (2.60%) | Glu (2.65%) | Glu (2.73%) |
| Gln (2.41%) | Gln (2.36%) | Gln (2.39%) | Gln (2.39%) | Gln (2.41%) | Gln (2.39%) | Gln (2.41%) | Gln (2.39%) | Gln (2.41%) |
| Arg (2.07%) | Arg (2.10%) | Asp (2.10%) | Asp (2.07%) | Arg (2.05%) | Lys (2.07%) | Lys (2.05%) | Lys (2.10%) | Lys (2.07%) |
| Asp (1.99%) | Lys (2.00%) | Arg (2.05%) | Arg (2.05%) | Asp (1.97%) | Arg (2.05%) | Arg (1.97%) | Arg (2.05%) | Arg (1.99%) |
| Lys (1.94%) | Asp (1.92%) | Lys (2.02%) | Lys (1.99%) | Lys (1.94%) | Asp (1.99%) | Asp (1.94%) | Asp (2.02%) | Asp (1.94%) |
| Cys (0.76%) | Cys (0.84%) | Cys (0.87%) | Cys (0.87%) | Cys (0.71%) | Cys (0.87%) | Cys (0.71%) | Cys (0.87%) | Cys (0.76%) |

| **Table S4.** The maximum (max.), mean and minimum (min.) p-distances between *Trichiurus* species (interspecific) and between genera within Trichiuridae (intergeneric) in each gene and mitogenome. | | | | | | | |  |
| --- | --- | --- | --- | --- | --- | --- | --- | --- |
|  | interspecific | | |  | intergeneric | | | |
|  | min. | mean | max. |  | min. | mean | max. | |
| 12S rRNA | 0.014 | 0.066 | 0.094 |  | 0.056 | 0.109 | 0.160 | |
| 16S rRNA | 0.015 | 0.054 | 0.077 |  | 0.054 | 0.112 | 0.181 | |
| ATP6 | 0.072 | 0.139 | 0.192 |  | 0.154 | 0.257 | 0.340 | |
| ATP8 | 0.042 | 0.139 | 0.196 |  | 0.257 | 0.369 | 0.470 | |
| COI | 0.054 | 0.104 | 0.132 |  | 0.139 | 0.168 | 0.192 | |
| COII | 0.036 | 0.103 | 0.153 |  | 0.151 | 0.195 | 0.242 | |
| COIII | 0.034 | 0.087 | 0.107 |  | 0.129 | 0.162 | 0.199 | |
| Cyt b | 0.072 | 0.121 | 0.143 |  | 0.154 | 0.211 | 0.243 | |
| ND1 | 0.054 | 0.138 | 0.168 |  | 0.144 | 0.194 | 0.243 | |
| ND2 | 0.070 | 0.138 | 0.160 |  | 0.151 | 0.200 | 0.253 | |
| ND3 | 0.063 | 0.140 | 0.174 |  | 0.203 | 0.235 | 0.266 | |
| ND4 | 0.069 | 0.140 | 0.177 |  | 0.152 | 0.213 | 0.266 | |
| ND4L | 0.037 | 0.128 | 0.168 |  | 0.154 | 0.195 | 0.230 | |
| ND5 | 0.057 | 0.130 | 0.164 |  | 0.172 | 0.170 | 0.344 | |
| ND6 | 0.073 | 0.189 | 0.241 |  | 0.165 | 0.247 | 0.311 | |
| Mitogenome* | 0.047 | 0.110 | 0.129 |  | 0.135 | 0.191 | 0.241 | |

* excluding d-loop.


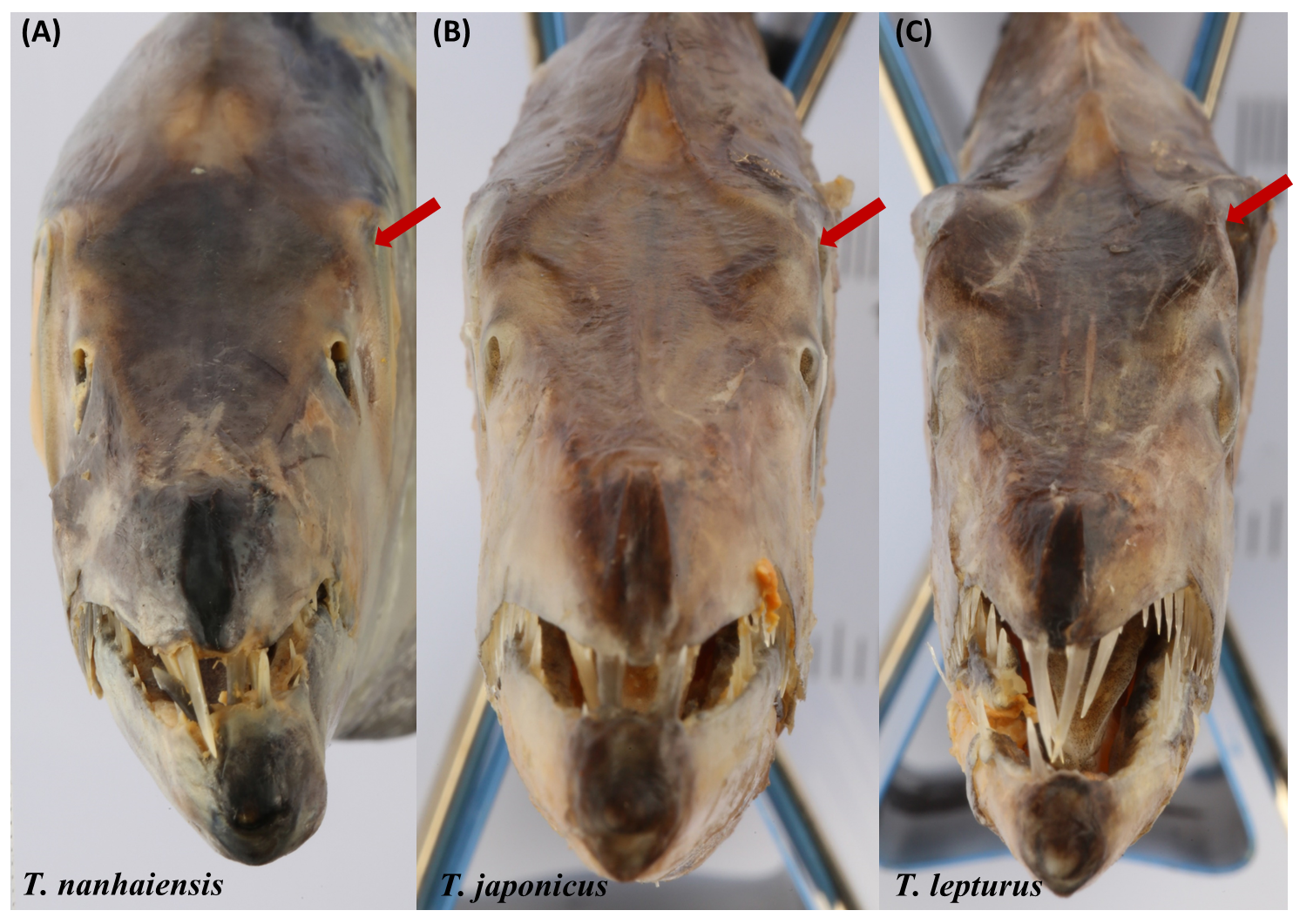


**Figure S1.** Frontal view of the heads of preserved specimens of *T. lepturus* species complex. **A** *T. nanhaiensis*, 83.4 cm TL, ZBL 000440, Zhanjiang; **B** *T. japonicus*, 85.6 cm TL, ZJ 1902, Zhanjiang; **C** *T. lepturus*, 76.3 cm TL, ZJ 1906, Zhanjiang.

**Figure S2.** The boxplot analyses in *T. japonicus* (blue), *T. lepturus* (orange), and *T. nanhaiensis* (grey). The landmarks are illustrated in Fig. 2.
